# Subtle effects of environmental stress observed in the early life stages of the Common frog, *Rana temporaria*

**DOI:** 10.1038/srep44438

**Published:** 2017-03-20

**Authors:** Rebecca Strong, Francis L. Martin, Kevin C. Jones, Richard F. Shore, Crispin J. Halsall

**Affiliations:** 1 Lancaster Environment Centre, Lancaster University, Bailrigg, Lancaster LA1 4YQ, UK; 2 School of Pharmacy and Biomedical Sciences, University of Central Lancashire (UCLan), Preston PR1 2HE, UK; 3 Centre for Ecology and Hydrology, Lancaster University, Bailrigg, Lancaster LA1 4YQ, UK

## Abstract

Worldwide amphibian populations are declining due to habitat loss, disease and pollution. Vulnerability to environmental contaminants such as pesticides will be dependent on the species, the sensitivity of the ontogenic life stage and hence the timing of exposure and the exposure pathway. Herein we investigated the biochemical tissue ‘fingerprint’ in spawn and early-stage tadpoles of the Common frog, *Rana temporaria*, using attenuated total reflection-Fourier-transform infrared (ATR-FTIR) spectroscopy with the objective of observing differences in the biochemical constituents of the respective amphibian tissues due to varying water quality in urban and agricultural ponds. Our results demonstrate that levels of stress (marked by biochemical constituents such as glycogen that are involved in compensatory metabolic mechanisms) can be observed in tadpoles present in the pond most impacted by pollution (nutrients and pesticides), but large annual variability masked any inter-site differences in the frog spawn. ATR-FTIR spectroscopy is capable of detecting differences in tadpoles that are present in selected ponds with different levels of environmental perturbation and thus serves as a rapid and cost effective tool in assessing stress-related effects of pollution in a vulnerable class of organism.

Globally, amphibians are facing precipitous declines^1^,^2^, with environmental pollution cited as a major threat to amphibian health and survival^3^,^4^. The vulnerability of amphibians to contaminant exposure is due to their highly permeable skin and complex lifecycle comprising both aquatic and terrestrial phases^5^,^6^,^7^. In addition, certain environmental contaminants, such as pesticides used in agriculture, are applied to adjacent land at the same time breeding and larval development occurs; a period thought to be particularly susceptible to the effects of chemical exposure^3^,^8^.

Monitoring amphibian populations from the same areas over time is of importance in order to track any deleterious changes that occur over multiple generations. Certain contaminants may also be maternally transferred to embryos following bioaccumulation throughout development, which may lead to impaired reproductive success^9^,^10^. As many anuran amphibian species show high breeding site fidelity and limited mobility between sites^11^,^12^, it is possible that the same populations may be monitored over time. Additionally, these factors may also mean that populations are susceptible to local extinctions, should environmental conditions change significantly^11^.

Amphibians, particularly at the aquatic stages of development are widely accepted as sensitive indicators of environmental contamination, and thus several studies have attempted to establish possible biomarkers of effect^3^,^13^. Endpoints commonly measured include growth^14^, behavioural abnormalities^15^, time to metamorphosis^14^,^16^, deformities^17^, endocrine disruption^8^,^18^,^19^, induction or suppression of enzymes and endogenous compounds related to oxidative metabolism^20^,^21^ suppression of immune function^22^,^23^ and genotoxicity^24^,^25^. While tadpoles at an early developmental stage are generally regarded as the stage most sensitive to environmental pollution^15^,^26^,^27^, amphibian embryos also show changes in developmental rates and subsequent deformities at metamorphosis as a result of earlier exposure to particular contaminants^27^,^28^,^29^, and thus it is important to consider this stage in any monitoring study.

Fourier-transform IR (FTIR) and attenuated total reflection-FTIR (ATR-FTIR) spectroscopy has previously been employed in order to identify potential biomarkers in fish^30^,^31^,^32^,^33^, birds^34^, and recently amphibians^35^, following exposure to contaminants both in the laboratory and field. Spectra derived using this approach represent a “biochemical cell fingerprint”, with wavenumbers corresponding to particular biochemical entities; such constituents include those related to the secondary structure of proteins (Amide I, II and III at ~1650 cm^−1^, ~1550 cm^−1^, ~1250 cm^−1^, respectively), lipids (~1750 cm^−1^), carbohydrates (~1150 cm^−1^ and ~1030 cm^−1^) and DNA/RNA (~1225 cm^−1^ and ~1080 cm^−1^)^36^. As the spectral data sets produced are typically large and complex, multivariate feature-extraction techniques such as principal component analysis (PCA) and linear discriminant analysis (LDA) are typically employed in order to reduce the data sets into less complex and more readily interpretable formats and identify which areas of the spectrum are responsible for differences between data sets^37^,^38^. Use of machine-learning techniques for classification of data also allows unknown samples to be classified on the basis of their IR spectra, and have previously been used to identify alterations induced by different pollutant types in bird feathers^34^, as well as the origin of oil spills from polluted beaches^39^.

The aim of this study was to determine whether stress, attributable to varying water quality, could be identified in spawn or early life-stage tadpoles (Gosner stages 25–28)^40^ of the common frog *Rana temporaria* using ATR-FTIR spectroscopy coupled with multivariate analysis and classification techniques over a three year period (2012–2014). The sites studied were in Northern England and were selected in order to give a comparison between a rural agricultural site with no pesticide input, a high pesticide-impacted agricultural site, and an urban site impacted by treated wastewater and landfill run-off. These sites are not subject to amphibian surveys and hence no time-series exists of population numbers, so the study carried out here could reveal the health-status of a given frog population with no *a priori* knowledge. Spawn and tadpoles were also compared annually at each pond in order to identify temporal differences in the spectral signatures generated.

Current work in biomedical science aims to use a spectroscopy-led approach to create a database of healthy individuals and those with diseases such as cancer in order to establish vibrational spectroscopy as a screening tool in disease diagnosis^38^,^41^,^42^. Whilst still only a relatively short-term monitoring study, the intention in this study was to ascertain a baseline level of ‘healthy’ amphibian embryos and tadpoles for a comparison with those from ponds with known water contamination. This approach could then potentially demonstrate the utility of vibrational spectroscopy as an environmental monitoring tool and identify amphibian populations affected by environmental perturbations such as water quality problems prior to any gross declines in population.

## Results

### Spatial differences between spawn samples

**Figure 1:**
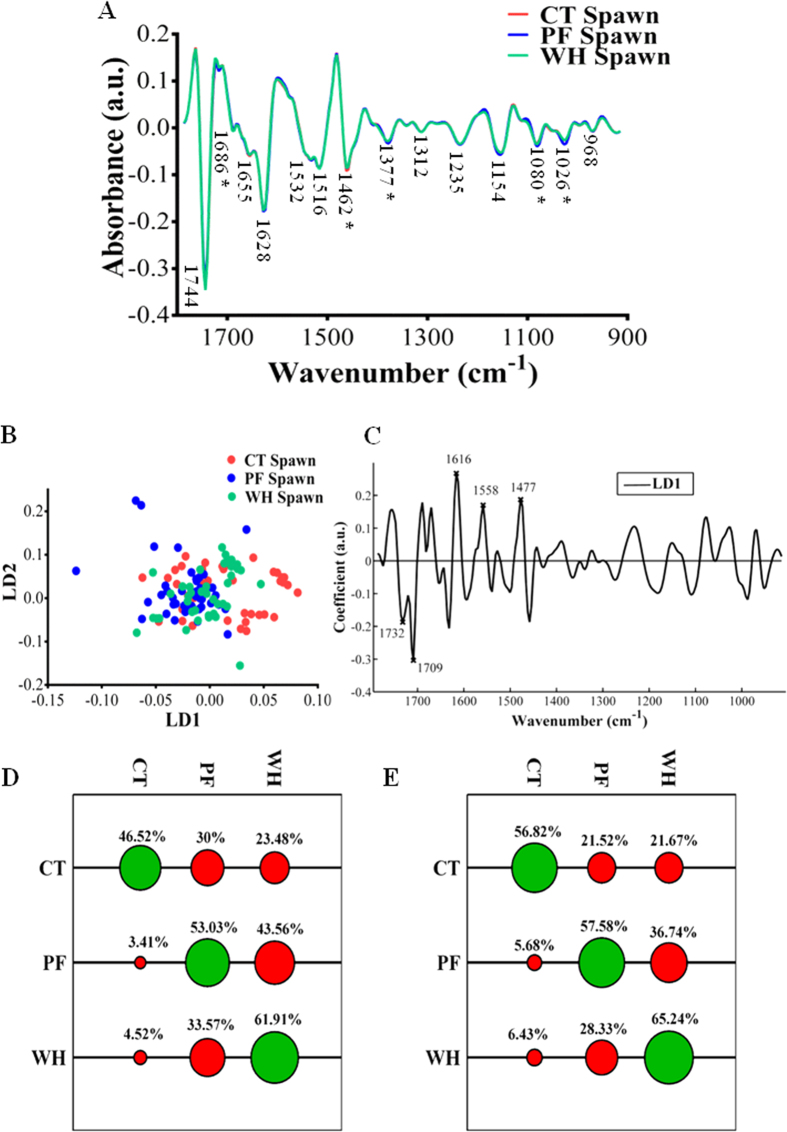
(**A**) Second derivative mean spectra of *Rana temporaria* spawn collected over a three year period (2012–2014) from CT (Crake Trees): a rural agricultural pond with minimal pesticide input; PF (Pennington Flash): an urban pond impacted by wastewater and landfill run-off and WH (Whinton Hill): an agricultural pond known to be impacted by pesticides. Spectra were cut at the biochemical fingerprint region (1800–900 cm^−1^), processed with Savitzky-Golay second-order differentiation and vector-normalised. Asterisks denote significant differences (*P* < 0.05) at absorbance peaks following one-way ANOVA. (**B**) Two-dimensional scores plot generated following cross-validated PCA-LDA analysis of spectra. (**C**) Corresponding loadings generated from PCA-LDA analysis; the five largest loadings values are highlighted. (**D**) Spawn classified by PCA-LDC. (**E**) Spawn classified by SVM. Green circles show % correct classification rate, red circles show % incorrect classification rate.

The mean spectra of spawn collected from each pond over the three-year period (*n* = ~45 spawn embryos per site) are shown in Fig. 1A. There is very little visual difference between the mean spectra of spawn collected from each site. Analysis with PCA-LDA followed by One-way ANOVA and Tukey’s multiple comparison tests demonstrated significant separation along LD1 between all three sites, but no separation along LD2 (Fig. 1B). The loadings from PCA-LDA demonstrated the regions attributable to the separation of spawn between ponds was predominantly in regions associated with protein (amide I and II regions) and C=O stretching of lipids (Fig. 1C, Table 1).

Further analysis of the peak heights revealed significant differences between spawn samples in regions associated with the following: Amide I proteins (1686 cm^−1^), with a larger peak in this region in spawn collected from WH (agricultural pond impact by pesticides and nutrient ions) in comparison to that collected from PF (urban pond receiving treated wastewater) or CT (agricultural pond with minimal pesticide input); CH_2_ stretching of lipids (1462 cm^−1^), with spawn samples from CT having a larger peak in this region in comparison to those from PF or WH; glycogen (1026 cm^−1^); this peak was significantly larger in spawn collected from PF in comparison to that from CT and WH, and a larger peak associated with CH_2_ symmetric bending modes of the methyl groups of proteins in spawn samples collected from CT and PF in comparison to those from WH (see Fig. 1A and Table 2). The classification of spawn based on pond of origin was generally quite poor for both PCA-LDC and SVM, the latter achieving a slightly higher classification rate (see Fig. 1D and E), although still only achieving correct classification up to a maximum of ~65% of the time for spawn collected from WH (Fig. 1E).

Comparisons were also made between spawn samples within each year group as shown in Supplementary Fig. 1A–F and Supplementary Table 1. Slightly clearer separation was seen in the scores plots of the spectra generated from spawn samples when analysed in this way, in particular in spawn samples collected in 2014. The main areas of the spectrum accounting for the separation between ponds each year were in regions associated with lipids (~1740 cm^−1^ and ~1460 cm^−1^) and proteins (~1650 cm^−1^), with come contribution from glycogen and symmetric phosphate (~1030 cm^−1^ and ~1080 cm^−1^ respectively).

### Spatial differences between tadpole samples

**Figure 2:**
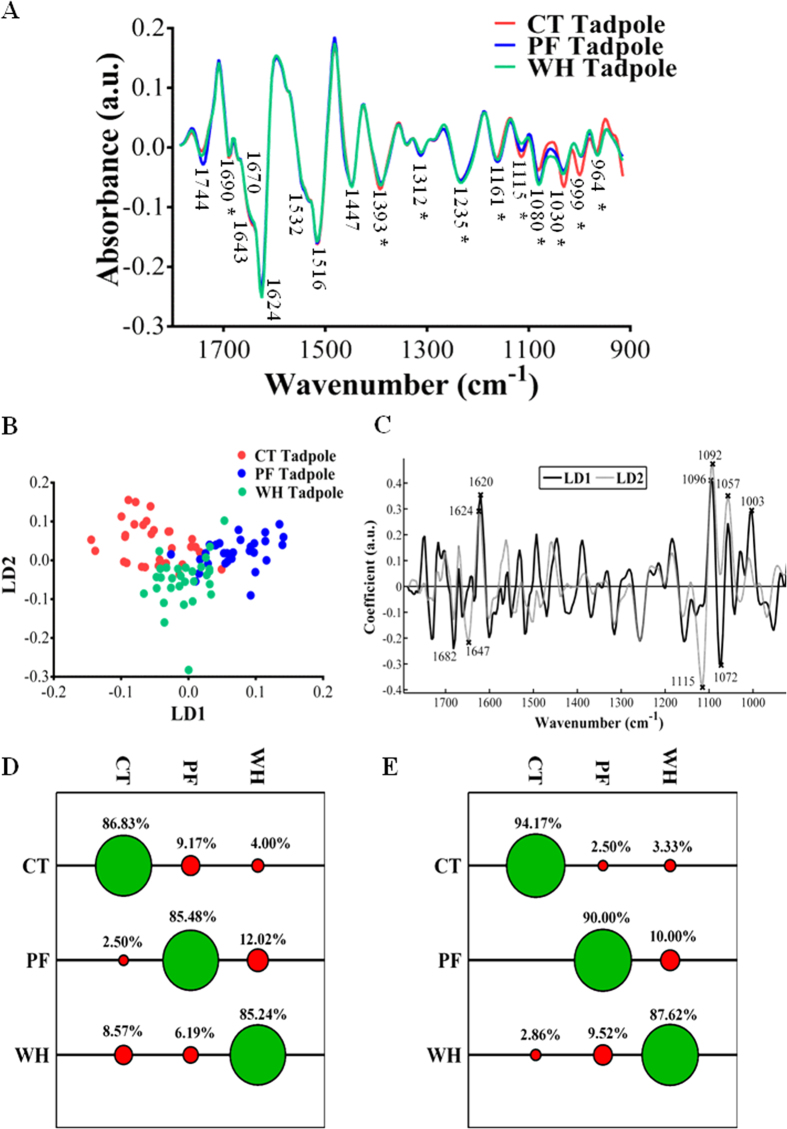
(**A**) Second derivative mean spectra of *Rana temporaria* tadpoles collected from ponds with differing water quality over a three year period (2012–2014) from CT: a rural agricultural pond with minimal pesticide input; PF: an urban pond impacted by wastewater and landfill run-off and WH: an agricultural pond known to be impacted by pesticides. Spectra were cut at the biochemical fingerprint region (1800–900 cm^−1^), processed with Savitzky-Golay second-order differentiation and vector-normalised. Asterisks denote significant differences (*P* < 0.05) at absorbance peaks following one-way ANOVA. (**B**) Two-dimensional scores plot generated following cross-validated PCA-LDA analysis of spectra. (**C**) Corresponding loadings generated from PCA-LDA analysis; the eight largest loadings values are highlighted. (**D**) Tadpoles classified by PCA-LDC. (**E**) Tadpoles classified by SVM. Green circles show % correct classification rate, red circles show % incorrect classification rate.

The mean spectra of tadpoles collected from each pond over a three year period (*n* = 90 (30 per site)) are shown in Fig. 2A. Visual inspection of the spectra suggests that some separation is apparent in the 1150–900 cm^−1^ region. Further analysis with PCA-LDA confirmed significant separation along LD1 between all three ponds (Fig. 2B) in regions associated predominantly with symmetric phosphate stretching vibrations of DNA/glycogen (~1100–1000 cm^−1^) as shown in the loadings plot in Fig. 2C. Additionally, regions associated with Amide I (proteins) also contributed to the separation along this dimension. LD2 accounts for separation between tadpoles collected from CT/PF and WH. This is again in similar regions as before: primarily symmetric phosphate stretching vibrations of DNA/glycogen (1092, 1057 cm^−1^) with some contribution from Amide I. The top five loadings values and corresponding wavenumber assignments are shown in Fig. 2C and Table 3.

Analysis of the peak absorbances confirmed significant differences in peak height primarily in the 1100–900 cm^−1^ phosphodiester region (see Table 2 and Fig. 2A), in particular in regions of the spectrum associated with glycogen (1030 cm^−1^) and C-C stretching of DNA (999 cm^−1^); these peaks were significantly larger in tadpoles collected from CT in comparison to those from PF and WH. There was no difference between tadpoles from PF and those from WH in these regions. In contrast, peak heights in regions associated with symmetric phosphate stretching vibrations (1080 cm^−1^) and C-C stretch of nucleic acids (964 cm^−1^) were significantly lower in tadpoles collected from CT in comparison to those from PF and WH; again there was no significant difference between tadpoles from PF and those from WH in this region. The region associated with P-O-C symmetric stretching (1115 cm^−1^) showed significant differences between tadpoles collected from all three sites in the order CT > PF > WH. In addition, there were significant differences in the peak associated with asymmetric stretching of phosphate (1235 cm^−1^), where tadpoles from WH showed increased absorbance in this area in comparison to those from CT and PF.

In contrast to the poor classification results achieved for spawn based on pond of origin, both PCA-LDC and SVM achieved high classification rates for tadpoles, demonstrating correct classification for tadpoles collected from each pond over 85% of the time (see Fig. 2D and E). SVM again achieved the highest classification rates, with tadpoles collected from CT correctly identified at the highest frequency, attaining a classification rate of ~94% (Fig. 2E). As shown in Supplementary Fig. 2, tadpoles did not differ in the majority of their body size measurements between ponds when all of the data were analysed together over the duration of the study, with the exception of HW, where tadpoles from PF had a significantly lower measurement of this parameter than those from CT (One-way ANOVA: *F*_*2, 87*_ = 3.97, *P* = 0.02; Tukey’s multiple comparison test, *P* < 0.05).

Comparisons between tadpoles from different ponds within each year group are shown in Supplementary Fig. 3A–F and Supplementary Table 2. In general, differences between tadpoles were in regions associated with carbohydrates, in particular glycogen (~1150 cm^−1^ and ~1030 cm^−1^), with significant contribution also from symmetric phosphate stretching of DNA and Amide I contributions. However, tadpoles collected in 2014 also showed significant lipid variation, with these differences mainly in the spectra of tadpoles collected from PF in comparison to those from CT and WH.

Significant differences in tadpole body size measurements were found between ponds, within each year group for tadpoles collected in 2013 and 2014, but not 2012 (see Supplementary Fig. 4), with tadpoles collected in 2013 from PF significantly smaller than those from both CT and WH on all measures of body size (One-way ANOVA: SVL: *F*_*2, 27*_ = 25.42, *P* < 0.001; HW: *F*_*2, 27*_ = 67.08, *P < *0.001; Mass: *F*_*2, 27*_ = 46.07, *P* < 0.001; Tukey’s multiple comparison tests, *P* < 0.05), but not BCI (BCI: *F*_*2, 27*_ = 1.12, *P* = 0.34). Tadpoles collected in 2014 from CT were smaller than those collected from PF on measures of SVL and mass, (One-way ANOVA: SVL: *F*_*2, 27*_ = 3.91, *P* = 0.03; Mass: *F*_*2, 27*_ = 6.09, *P* = 0.007; Tukey’s multiple comparison tests, *P* < 0.05). Tadpoles from WH also had a smaller mass in comparison to those from PF in 2014 (Tukey’s multiple comparison tests, *P* < 0.05).

As significant differences in body size measurements were found between tadpoles from PF and those from CT and WH in 2013 and 2014, separate analysis was conducted with tadpoles from PF excluded from the analysis in order to remove the potentially confounding effects of body size. Significant differences were apparent between tadpoles from CT and those from WH in 2013 along PCs 2 and 3 in regions associated with C=O stretching of lipids, amide I proteins and symmetric stretching of P-O-C and nucleic acids (Supplementary Fig. 5, Supplementary Table 3). In 2014, there were differences between tadpoles from CT and those WH along PC2 only, in regions associated predominantly with carbohydrates/glycogen and sugar phosphate vibrations in nucleic acids, with some lipid contribution.

### Temporal differences

As annual differences in environmental conditions may affect amphibian health and development, comparisons between spawn and tadpole samples between years within each pond were also made, to determine if these differences were expressed in consistent areas of the spectrum. Differences in body size measurements between tadpoles were also determined. Maximum, minimum and average air temperatures were obtained each year and details are provided in Supplementary Fig. 6, with clear differences apparent each year with average, minimum and maximum temperatures lower in 2013 in February/March in comparison to 2012 and 2014, which is reflected in the variation in spawning date and tadpole development shown in Supplementary Tables 4 and 5, with spawning and tadpole development occurring up to 6 weeks later in 2013 than in 2012 and 2014.

Results from the analysis of spectra generated from spawn between years within each pond are shown in Supplementary Fig. 7A–F and Supplementary Table 6. It is clear that much better separation is seen in the scores plots of the spectra generated from spawn samples when analysed in this way. The areas of the spectrum accounting for the separation of spectra generated from spawn samples between years show significant overlap with those from the spectra generated between ponds, as determined by the loadings plots in Supplementary Fig. 7. Again, similar to the differences seen in the spectra of spawn samples between ponds, the differences in the spectra of spawn samples between years were primarily in regions associated with C=O stretching of lipids (1744 cm^−1^) and Amide I proteins (~1700–1600 cm^−1^), with most separation apparent between spawn samples collected in 2012 and those collected in 2014.

Comparisons between tadpole samples between years within each pond are shown in Supplementary Fig. 8A–F and Supplementary Table 7. Significant separation was also seen when the spectra were analysed in this way; however the differences between the spectra generated from tadpoles were primarily in regions associated with lipids, in particular the C=O stretching and CH_2_ scissoring mode of the acyl chain of lipid (~1740 and ~1460 cm^−1^), with some protein contribution, in contrast to the differences seen in the spectra of tadpoles between ponds, which were mainly in the phosphodiester region of the spectrum (~1150–900 cm^−1^). Similar to the temporal differences seen in the analysis of spawn samples, the largest differences appeared to be between tadpoles collected in 2012 and those collected in 2014.

Significant differences in tadpole body size measurements within ponds between different years were also found as shown in Supplementary Fig. 9. Tadpoles collected in 2014 from both CT and WH were smaller than those collected in 2013 or 2014 on most measures of body size (One-way ANOVA: CT: SVL: *F*_*2, 27*_ = 18.50, *P* < 0.001; HW: *F*_*2, 27*_ = 19.47, *P* < 0.001; Mass: *F*_*2, 27*_ = 19.45, *P* < 0.001; Tukey’s multiple comparison tests, *P* < 0.05: WH: SVL: *F*_*2, 27*_ = 8.22, *P* = 0.002; HW: *F*_*2, 27*_ = 12.23, *P* < 0.001; Mass: *F*_*2, 27*_ = 16.85, *P* < 0.001) whereas tadpoles collected from PF collected in 2013 were generally smaller than those collected in 2012 or 2014 (One-way ANOVA: SVL: *F*_*2, 27*_ = 11.31, *P* < 0.001; HW: *F*_*2, 27*_ = 15.78, *P* < 0.001; Mass: *F*_*2, 27*_ = 10.13, *P* = 0.001; Tukey’s multiple comparison tests, *P* < 0.05). Body condition indices were only lower in tadpoles collected from CT in 2012 in comparison to those in 2013 and 2013 (BCI: *F*_*2, 27*_ = 4.80, *P* = 0.02; Tukey’s multiple comparison tests, *P* < 0.05), but not at PF or WH.

## Discussion

Amphibians are sensitive to environmental pollution due to their life history and a tendency to show high site fidelity, thus allowing repeated exposure to environmental contaminants over time^11^. Although species such as *R. temporaria* are relatively abundant^43^, they may serve as a useful sentinel species in environmental monitoring studies as a proxy for rarer species. This study has demonstrated that ATR-FTIR spectroscopy in conjunction with multivariate analysis and classification techniques is able to effectively distinguish between tadpoles of the common frog, *R. temporaria* collected from three ponds with differing water quality over a three-year period. This was in spite of annual differences, which were also apparent when the data were analysed each year. In contrast, the differences between years for spawn were much more profound than those between ponds, suggesting that annual differences masked many of the differences detected in the IR spectra of spawn collected from each pond.

In this study, there were minimal differences in body size between tadpoles (with the exception of head width between PF and CT tadpoles), when all of the data were analysed together, thus excluding body size as a reason for the separation and high classification rates seen between ponds. The differences between ponds were largely in areas associated with glycogen/carbohydrates and symmetric phosphate stretching, with some protein contribution. Glycogen, and to a lesser extent, protein, is utilised as an energy source in amphibians and may be depleted in response to stressful situations, such as exposure to environmental contaminants, as the organism attempts to maintain homeostasis by compensatory metabolic mechanisms, thus utilising energy reserves^44^,^45^. Regions of the IR spectrum associated with carbohydrates, particularly glycogen showed marked decreases in the peak heights in spectra of tadpoles from PF and WH (both sites with relatively lower water quality) in comparison to those from CT (higher water quality status). Several studies have measured glycogen levels in tissues of both larval and adult amphibians following exposure to various environmental contaminants, including pesticides such as atrazine^44^,^46^,^47^, glyphosate^44^, quinclorac^44^, basudin^48^, naphthenic acids^45^ and PAHs^49^. In general, these studies found depleted levels of glycogen in response to pesticide exposure, although not in all cases^46^.

The increases in asymmetric and symmetric phosphate stretching vibrations absorbance seen in the spectra of tadpoles from WH and to a lesser extent PF, may be reflective of the type of contaminants tadpoles were exposed to as the ponds studied were subject to run-off from agricultural and urban environments^35^, which may be associated with genotoxicity^7^,^25^. Previous studies utilising IR spectroscopy to assess the health of fish following exposure to environmental contaminants have also demonstrated a pattern of decreased glycogen absorbance and increases in asymmetric and symmetric phosphate following exposure to environmental contaminants such as endocrine disruptors^32^,^50^ and PAHs^31^, as found in this study. However caution must be exercised in interpreting the results as by the nature of the study, tadpoles were exposed to a mixture of xenobiotics as well as varying nutrient levels and no one single factor can be elucidated.

The differences between years for both tadpoles and spawn are unsurprising given the factors that may vary each year, such as temperature, and therefore date of spawning, food availability, competition and predation. Interestingly, the differences seen between tadpoles from different years were in different areas of the spectrum in comparison to the differences seen between tadpoles from different ponds. Between ponds, tadpoles varied in regions associated with carbohydrates and asymmetric and symmetric phosphate stretching with some protein contribution, whereas between years the differences were mainly confined to areas of the spectrum associated with lipids and proteins (mainly Amide I and II). These differences may be tied to body size differences, as there was variability in tadpole body size parameters between years within each site. Tadpoles show developmental plasticity, where they are able to adjust their developmental rate according to environmental conditions, producing smaller individuals under conditions of low food availability and high population density^51^,^52^,^53^,^54^. Although there were body size differences between tadpoles from PF and those from CT/WH in 2013/4, once tadpoles from PF were excluded from the analysis (thus excluding body size as a confounding factor) there was still significant separation between tadpoles from CT and those from WH in spectral regions associated with amide I proteins, symmetric phosphate stretching and carbohydrates/glycogen.

In biomedical studies involving disease screening, there naturally exists variation between individuals and possible confounding variables between samples^55^,^56^,^57^. Therefore screening programmes using spectroscopy must be specific enough to determine signatures attributable to a particular disease state in spite of ‘noise’ in the data. Chemometric processing of the data, often using multivariate methods is thus an important step in distinguishing between ‘healthy’ and ‘diseased’ tissues in these highly complex data sets. In addition, patients are matched for potentially confounding factors such as age or ethnicity where possible^58^. In this study, it appears that tadpole body size may influence biochemical parameters as determined by the IR spectra generated. Therefore, as with biomedical studies, it is recommended that any future study should ideally case-match tadpoles on the basis of their body size, developmental stage and where possible abiotic factors, such as temperature, pH and dissolved oxygen in order to control for such factors.

In contrast to the clear spectral differences seen between tadpoles, the differences between spawn samples between ponds were in similar areas of the spectrum to those between years, being predominantly in areas associated with protein and lipids. This may account for the poorer separation and classification seen in spawn samples in comparison to tadpoles. There are several factors influencing the development of spawn including temperature, oxygen levels and maternal investment^59^,^60^,^61^,^62^. Unfortunately these factors cannot be controlled for in a field study of this kind. Temperature is capable of influencing egg development markedly, with date of spawning significantly correlated with ambient water temperature^59^,^60^. Indeed, there were differences seen in this study in terms of date of spawning, with frogs spawning in early/mid March in 2012 and 2014 (between 7^th^ and 16^th^ March), whereas this was delayed in 2013 to late March/early April in 2013, which was likely related to temperature, as average, minimum and maximum temperatures were lower around this time in 2013. Additionally in 2012, maximum temperatures were higher around the times of spawning in comparison to 2013 and 2014, which again may have influenced spawn development, with a reduction in clutch fecundity associated with extreme temperatures in the preceding year^59^. Amphibian embryos are also protected from xenobiotics by the jelly capsule surrounding the embryo^15^,^26^. This may also explain why the differences detected between embryos in the current study were relatively smaller in comparison to that of the tadpoles despite water quality differences between the sites.

This study demonstrated the use of ATR-FTIR spectroscopy as a monitoring tool in assessing the health of *R. temporaria* spawn and tadpoles from three ponds with relative differences in water quality. This technique therefore offers a unique method to assess the stress status of wild populations living in contaminated sites. Tadpoles at an early stage in development demonstrated the most significant differences in their IR spectra and are thus proposed as a more sensitive life stage for spectroscopic assessment of environmental quality. With complementary laboratory and mesocosm studies, IR spectroscopy could be a highly useful, cost-effective and rapid tool in monitoring amphibian health. In addition, the use of hand-held IR devices could potentially allow the non-destructive monitoring of amphibians throughout their development. Field-based FTIR devices for this type of analysis could provide rapid insight into the biochemical status of different tissue types with minimal sample preparation or processing, providing insight into the health status of a given population which could be of great benefit to the many species of amphibian vulnerable to extinction.

## Methods

### Field Sites

Sites were selected in order to give a comparison between agricultural and urban ponds and were based on site characteristics and information from landowners/land managers. The sites were:

1. Whinton Hill (WH), Plumpton, Cumbria is a farm consisting of arable and grazing land for beef and sheep, which is routinely sprayed with herbicides and fungicides.
2. Crake Trees (CT), Crosby Ravensworth is a farm used as beef grazing land and marginal arable land, which has been accepted onto Natural England’s Higher Level Environmental Stewardship Scheme and uses minimal quantities of pesticides, with buffer zones to prevent pesticide run-off into water courses. The ponds surveyed at WH and CT are constructed wetlands created as part of the MOPS2 (Mitigation Options for Phosphorus and Sediment) project monitored by Lancaster University http://mops2.diffusepollution.info/.
3. Pennington Flash Country Park (PF) located in Leigh, Lancashire is a site which receives run-off from treated wastewater and landfill, as this area was previously a landfill site.

Water quality for each pond was assessed through the measurement of key ions (including nitrate (NO_3_-N) and phosphate (PO_4_-P) as well as a range of organic chemical pollutants including a broad screen of current–use pesticides. A summary of concentrations are presented in Strong *et al*.^35^. In brief, water quality with respect to these chemical parameters measured during Spring months resulted in the ranking of the ponds as: CT highest water quality, followed by PF with WH having the lowest water quality of the three ponds.

### Collection and processing of samples

Samples of *R. temporaria* spawn were collected in 2012, 2013 and 2014 (*n* = ~135 in total) from all three sites (10–20 spawn embryos per site per year), at varying dates depending on the date of spawning (full details in Supplementary Table 4). Spawn was collected in solvent-rinsed glass jars and transported back to the laboratory before the jelly coat was removed with forceps and the embryo fixed in 70% ethanol overnight at 4 °C. The Gosner stage of spawn samples was noted prior to fixation^40^. Spawn was classified as Gosner stage 10–12. Whole fixed embryos were mounted directly onto Low-E reflective glass slides (Kevley Technologies, Chesterland, OH, USA), dried overnight and stored in a desiccator before subsequent interrogation with ATR-FTIR spectroscopy.

*Rana temporaria* tadpoles were caught from all three sites in 2012, 2013 and 2014 (*n* = 90 in total) using dip nets (ten per site per year), euthanised using a solution of MS-222 (200 mg/L) buffered with sodium bicarbonate (both from Sigma Aldrich, Poole, Dorset UK), as per Schedule 1 of the British Home Office Animals (Scientific Procedures) Act 1986. Tadpole samples were then rinsed in distilled water and fixed immediately in the field in 70% ethanol (Fisher Scientific, UK). Ethanol was replaced after 24 hours with fresh. Tadpoles were weighed and measurements taken of snout-vent length (SVL) and head width (HW) using digital callipers to the nearest 0.01 mm after fixation. Tadpoles were staged according to Gosner (1960), with all tadpoles between stages 25–28. Body condition indices (BCI) were calculated for each tadpole as follows: (body mass/SVL^3^) × 100^45^ (full details of Gosner stage, SVL, HW, body mass and BCI for each tadpole are provided in Supplementary Table 5).

For ATR-FTIR spectroscopy measurements, a longitudinal slice (~0.5 mm thick) was taken from the ventral side of the tadpole using a Stadie-Riggs tissue slicer; a simple technique previously employed for preparing tissue samples for analysis with IR spectroscopy^31^,^63^,^64^. Slices were mounted skin side down onto Low-E slides, dried overnight and stored in a desiccator before interrogation with ATR-FTIR spectroscopy.

### Temperature data

Temperature data (maximum, minimum and average air temperatures) were obtained from the Hazelrigg weather station at Lancaster University covering two week time periods beginning approximately one month prior to the start of the breeding season (~29^th^ January) and finishing after all individuals had gone through metamorphosis (~26^th^ August) for each year. Details are provided in Supplementary Fig. 6.

### ATR-FTIR Spectroscopy

Between 10 and 25 spectra were taken per sample of spawn and tadpole using a Tensor 27 FTIR spectrometer with Helios ATR attachment (Bruker Optics Ltd, Coventry, UK) containing a diamond crystal (≈250 μm × 250 μm sampling area). Spectra were acquired at 8 cm^−1^ resolution with 2× zero-filling, giving a data-spacing of 4 cm^−1^ over the range 400–4000 cm^−1^. The crystal was cleaned with distilled water between the analysis of each sample and a new background reading was taken prior to the analysis of each sample in order to account for changes in atmospheric conditions.

### Data pre-processing

A representative ATR-FTIR spectrum was obtained by taking the mean of the spectral measurements for each sample. Spectra were then cut at the biochemical cell fingerprint region (1800–900 cm^−1^), baseline corrected using Savitzky-Golay 2^nd^ order differentiation (2^nd^ order polynomial and 9 filter coefficients), and vector normalised.

### Multivariate analysis

Data were mean-centred before input into principal component analysis-linear discriminant analysis (PCA-LDA) with *k*-folds cross validation, where *k* = 5; this method uses a small portion of the dataset to train the model in order to prevent LDA overfitting^37^. PCA reduces the spectra (227 wavenumbers) into a smaller number of principal components for input into LDA. In this case 9 PCs were picked for spawn analysis and 12 for analysis of tadpoles, using the PCA Pareto function in the IRootLab toolbox, as this represented ~95% of the variance in the data and where the variance began to plateau, thus preventing noise being incorporated into the LDA algorithm. LDA maximises the differences between classes and minimises the heterogeneity within classes. The data can then be viewed as scores, to determine how the different classes separate from each other. The corresponding loadings vectors when viewed alongside the scores allow the wavenumbers which contribute maximally to the variance to be identified^37^.

For both analysis of spawn and tadpoles, data were classed by pond (CT, PF and WH) using all of the data collected over the three-year period. This was the main goal of the study; identifying differences between ponds despite annual variations. Additionally, samples of spawn and tadpoles were analysed within each year group using PCA alone due to the reduced sample size^38^,^65^ to determine if the differences between ponds were consistently expressed each year. Within each pond, annual differences were also determined to identify which, if any areas of the spectrum corresponded to annual factors. Finally, as tadpole body size parameters showed a large variation over the course of the study (see Supplementary Fig. 4, with raw data in Supplementary Table 5), with significant variation found between tadpoles from PF and those from CT and WH in 2013 and 2014, separate analysis was conducted between tadpoles from CT and those from WH, excluding tadpoles from PF to try and exclude the effect of body size on the results.

### Classification of data

For this study, two commonly applied classifiers; principal component analysis-linear discriminant classifier (PCA-LDC) and support vector machines (SVMs) were employed for comparison of their classification ability. Both are supervised classification techniques i.e. where the classes are labelled *a priori.* PCA-LDC is used for linear classification, whereas SVM has the advantage of being able to separate data which do not follow a linear pattern. The output from each classifier was a ‘classification accuracy rate’, which is defined as the average between sensitivity (true positives) and specificity (true negatives)^66^. Full details of the theory behind each technique are provided in the Supplementary Note 1.

All spectral pre-processing and data analysis was implemented using the IRootLab toolbox http://trevisanj.github.io/irootlab/67,68 in Matlab (r2012a) (The MathWorks, Inc., USA), unless otherwise stated.

### Statistical analysis

One-way ANOVA followed by Tukey’s multiple comparison tests, or two-sample t-tests where appropriate, were conducted to determine significant differences between classes using the scores from the PCA-LDA and PCA outputs. One-way ANOVA followed by Tukey’s multiple comparison tests were also used to determine significant differences between body size parameters. One-way ANOVA was also used to determine differences in the absorbance values of the second derivative spectra (full details in Supplementary Note 2). These analyses were conducted in XLSTAT (Addinsoft, Paris).

## Additional Information

**How to cite this article:** Strong, R. *et al*. Subtle effects of environmental stress observed in the early life stages of the Common frog, *Rana temporaria. Sci. Rep.* **7**, 44438; doi: 10.1038/srep44438 (2017).

**Publisher's note:** Springer Nature remains neutral with regard to jurisdictional claims in published maps and institutional affiliations.

## Supplementary Material

Supplementary Information

## Figures and Tables

**Table 1:** Distinguishing wavenumbers and proposed assignments obtained from analysis of *Rana temporaria* spawn with ATR-FTIR spectroscopy following analysis with PCA-LDA.

Comparison	Wavenumber (cm^−1^)	Tentative Assignment^¥^	Comparison^≠^
By site LD1	1732	C=O stretching of lipids	CT^a^
	1709	C=O stretching (bases)	PF^b^
	1616	Amide I (carbonyl stretching vibrations in side chains of amino acids)	WH^c^
	1558	Amide II proteins	
	1477	CH_2_ lipids	

The five largest loadings values for significant linear discriminants (LD) are shown. Comparisons were made between sites: CT (Crake Trees): a rural agricultural pond with no pesticide input; WH (Whinton Hill): an agricultural pond known to be impacted by pesticides and PF (Pennington Flash): an urban pond impacted by wastewater and landfill run-off.

^¥^Refs 36, 69 and 70.

^≠^Different letters denote a significant difference at the *P* < 0.05 level following one-way ANOVA and Tukey’s multiple comparison tests.

**Table 2:** Wavenumbers and assigned bands of infrared peaks following ATR-FTIR analysis of spawn and whole tadpoles of *Rana temporaria*.

Life Stage	Wavenumber (cm^−1^)	Proposed Assignment^a^	Comparison
Spawn	1744	C=O stretching of lipids	CT = PF
			CT = WH
			PF = WH
	1686	Amide I (Intermolecular *β*-sheet)	CT = PF
			**CT** < **WH**
			**PF** < **WH**
	1655	Amide I of proteins (α helix)	CT = PF
			CT = WH
			PF = WH
	1628	Amide I (Intramolecular *β*-sheet)	CT = PF
			CT = WH
			PF = WH
	1532	Amide II, C≡N stretching	CT = PF
			CT = WH
			PF = WH
	1516	Amide II of proteins	CT = PF
			CT = WH
			PF = WH
	1462	CH_2_ stretching of lipids	**CT** > **PF**
			**CT** > **WH**
			PF = WH
	1377	CH_2_ symmetric bending modes of the methyl groups of proteins	CT = PF
			**CT** > **WH**
			**PF** > **WH**
	1312	Amide III of proteins	CT = PF
			CT = WH
			PF = WH
	1235	PO_2_^−^ asymmetric stretching, with overlap from Amide III	CT = PF
			CT = WH
			PF = WH
	1154	Stretching vibrations of hydrogen-bonded C-OH groups	CT = PF
			CT = WH
			PF = WH
	1080	PO_2_^−^ symmetric stretching vibrations: nucleic acids and phospholipids	**CT** < **PF**
			CT = WH
			PF = WH
	1026	Glycogen absorption (C-O stretching)	**CT** < **PF**
			CT = WH
			**PF** > **WH**
	968	C-C DNA	CT = PF
			CT = WH
			PF = WH
			
Tadpole	1744	C=O stretching of lipids	CT = PF
			CT = WH
			PF = WH
	1690	Peak of nucleic acids due to carbonyl stretching	CT = PF
			CT = WH
			**PF** < **WH**
	1670	Amide I (anti-parallel *β*-sheet)	CT = PF
			CT = WH
			PF = WH
	1643	Amide I (C=O vibrations)	CT = PF
			CT = WH
			PF = WH
	1624	Amide I, *β*-sheet	CT = PF
			CT = WH
			PF = WH
	1532	Amide II, C≡N stretching	CT = PF
			CT = WH
			PF = WH
	1516	Amide II of proteins	CT = PF
			CT = WH
			PF = WH
	1447	CH_2_ bending of lipids and fatty acids	CT = PF
			CT = WH
			PF = WH
	1393	CH_3_ bending of proteins and lipids	**CT** > **PF**
			CT = WH
			PF = WH
	1312	Amide III of proteins	**CT** < **PF**
			CT = WH
			**PF** > **WH**
	1235	PO_2_^−^ asymmetric stretching vibrations	CT = PF
			**CT** < **WH**
			**PF** < **WH**
	1161	C-O stretching	**CT** < **PF**
			CT = WH
			PF = WH
	1115	Symmetric stretching P-O-C	**CT** > **PF**
			**CT** > **WH**
			**PF** > **WH**
	1080	PO_2_^−^ symmetric stretching vibrations: nucleic acids and phospholipids	**CT** < **PF**
			**CT** < **WH**
			PF = WH
	1030	Glycogen vibration	**CT** > **PF**
			**CT** > **WH**
			PF = WH
	999	C-C vibration of DNA	**CT** > **PF**
			**CT** > **WH**
			PF = WH
	964	C-C stretch of nucleic acids	**CT** < **PF**
			**CT** < **WH**
			PF = WH

Absorbance values of second derivatives were compared between CT: (Crake Trees) a rural agricultural pond with no pesticide input; WH: (Whinton Hill) an agricultural pond known to be impacted by pesticides and PF: (Pennington Flash) an urban pond impacted by wastewater and landfill run-off. Significance was calculated at the *P* < 0.05 level for spawn and tadpoles following one-way ANOVA and Tukey’s multiple comparison tests. Significant results are in bold.

^a^Refs 32, 36, 50, 69, 71 and 72.

**Table 3:** Distinguishing wavenumbers and proposed assignments obtained from analysis of Rana temporaria tadpoles with ATR-FTIR spectroscopy following analysis with PCA-LDA.

Comparison	Wavenumber (cm^−1^)	Tentative Assignment^¥^	Comparison^≠^
LD1	1682	Amide I deformation	CT^a^
	1620	Peak of nucleic acids due to the base carbonyl stretching and ring breathing mode	PF^b^
	1096	Stretching PO_2_^−^ symmetric vibrations	WH ^c^
	1072	Nucleic acid band (symmetric phosphate stretch)	
	1003	Sugar phosphate chain vibrations in nucleic acids	
LD2	1647	Amide I	CT^a^
	1624	Amide I, *β*-sheet	PF^a^
	1115	Symmetric stretching P-O-C	WH^b^
	1092	Symmetric phosphate stretching	
	1057	Glycogen	

The five largest loadings values for the two linear discriminants (LD) are shown. Comparisons were made between sites: CT: a rural agricultural pond with no pesticide input; WH: an agricultural pond known to be impacted by pesticides and PF: an urban pond impacted by wastewater and landfill run-off.

^¥^Refs 32, 33, 36 and 72.

^≠^Different letters denote a significant difference at the *P* < 0.05 level following one-way ANOVA and Tukey’s multiple comparison tests.

## References

[b1] Alford R. A. & Richards S. J. Global amphibian declines: a problem in applied ecology. Annual review of Ecology and Systematics 133–165 (1999).

[b2] Wake D. B. & Vredenburg V. T. Are we in the midst of the sixth mass extinction? A view from the world of amphibians. Proceedings of the National Academy of Sciences 105, 11466–11473 (2008). 10.1073/pnas.0801921105 PMC2556420 18695221

[b3] Mann R., Hyne R., Choung C. & Wilson S. Amphibians and agricultural chemicals: Review of the risks in a complex environment. Environmental Pollution 157, 2903–2927, doi: 10.1016/j.envpol.2009.05.015 (2009). 19500891

[b4] Carey C. & Bryant C. J. Possible interrelations among environmental toxicants, amphibian development, and decline of amphibian populations. Environmental Health Perspectives 103, 13 (1995). 10.1289/ehp.103-1519280 PMC1519280 7556018

[b5] Brühl C. A., Pieper S. & Weber B. Amphibians at risk? Susceptibility of terrestrial amphibian life stages to pesticides. Environmental Toxicology and Chemistry 30, 2465–2472, doi: 10.1002/etc.650 (2011). 21898550

[b6] Cooke A. S. Tadpoles as indicators of harmful levels of pollution in the field. Environmental Pollution Series A, Ecological and Biological 25, 123–133, doi: 10.1016/0143-1471(81)90012-x (1981).

[b7] Ralph S. & Petras M. Caged amphibian tadpoles and *in situ* genotoxicity monitoring of aquatic environments with the alkaline single cell gel electrophoresis (comet) assay. Mutation Research-Genetic Toxicology and Environmental Mutagenesis 413, 235–250, doi: 10.1016/s1383-5718(98)00024-2 (1998). 9651536

[b8] Hayes T. B. . Pesticide mixtures, endocrine disruption, and amphibian declines: Are we underestimating the impact? Environmental Health Perspectives 114, 40–50, doi: 10.1289/ehp.8051 (2006). PMC1874187 16818245

[b9] Bergeron C. M., Bodinof C. M., Unrine J. M. & Hopkins W. A. Bioaccumulation and maternal transfer of mercury and selenium in amphibians. Environmental Toxicology and Chemistry 29, 989–997, doi: 10.1002/etc.125 (2010). 20821530

[b10] Todd B. D., Bergeron C. M., Hepner M. J. & Hopkins W. A. Aquatic and terrestrial stressors in amphibians: A test of the double jeopardy hypothesis based on maternally and trophically derived contaminants. Environmental Toxicology and Chemistry 30, 2277–2284, doi: 10.1002/etc.617 (2011). 21755529

[b11] Blaustein A. R., Wake D. B. & Sousa W. P. Amphibian declines: judging stability, persistence, and susceptibility of populations to local and global extinctions. Conservation Biology 8, 60–71 (1994).

[b12] Laurila A. & Aho T. Do female common frogs choose their breeding habitat to avoid predation on tadpoles? Oikos 585–591 (1997).

[b13] Venturino A. . Biomarkers of effect in toads and frogs. Biomarkers 8, 167–186, doi: 10.1080/1354700031000120116 (2003). 12944171

[b14] Relyea R. A. & Diecks N. An unforeseen chain of events: Lethal effects of pesticides on frogs at sublethal concentrations. Ecological Applications 18, 1728–1742, doi: 10.1890/08-0454.1 (2008). 18839767

[b15] Cooke A. S. The effects of DDT, dieldrin and 2,4-D on amphibian spawn and tadpoles. Environmental Pollution 3, 51–68, doi: 10.1016/0013-9327(72)90017-1 (1972).

[b16] Greulich K. & Pflugmacher S. Differences in susceptibility of various life stages of amphibians to pesticide exposure. Aquatic Toxicology 65, 329–336, doi: 10.1016/s0166-445x(03)00153-x (2003). 13678851

[b17] Ruiz A. M. . Patterns of development and abnormalities among tadpoles in a constructed wetland receiving treated wastewater. Environmental Science & Technology 44, 4862–4868, doi: 10.1021/es903785x (2010). 20540544

[b18] Harris M. L., Bishop C. A. & McDaniel T. V. Assessment of rates of deformity in wild frog populations using *in situ* cages: a case study of Leopard Frogs (*Rana pipiens*) in Ontario, Canada. Biomarkers 6, 52–63, doi: 10.1080/135475001452797 (2001). 23886057

[b19] Gabor C. R., Zabierek K. C., Kim D. S., Alberici da Barbiano L., Mondelli M. J., Bendik N. F. & Davis D. R. A non-invasive water-borne assay of stress hormones in aquatic salamanders. Copeia 104, 172–181 (2016).

[b20] Ferrari A., Lascano C. I., Anguiano O. L., D’Angelo A. M. P. d. & Venturino A. Antioxidant responses to azinphos methyl and carbaryl during the embryonic development of the toad *Rhinella (Bufo) arenarum* Hensel. Aquatic Toxicology 93, 37–44 (2009). 19362380 10.1016/j.aquatox.2009.03.003

[b21] Lajmanovich R. C. . Activity levels of *β*-esterases in the tadpoles of 11 species of frogs in the middle Parana River floodplain: Implication for ecological risk assessment of soybean crops. Ecotoxicology and Environmental Safety 73, 1517–1524, doi: 10.1016/j.ecoenv.2010.07.047 (2010). 20708801

[b22] Carey C., Cohen N. & Rollins-Smith L. Amphibian declines: an immunological perspective. Developmental and Comparative Immunology 23, 459–472, doi: 10.1016/s0145-305x(99)00028-2 (1999). 10512457

[b23] Christin M. S. . Effects of agricultural pesticides on the immune system of *Xenopus laevis* and *Rana pipiens*. Aquatic Toxicology 67, 33–43, doi: 10.1016/j.aquatox.2003.11.007 (2004). 15019249

[b24] Clements C., Ralph S. & Petras M. Genotoxicity of select herbicides in *Rana catesbeiana* tadpoles using the alkaline single-cell gel DNA electrophoresis (comet) assay. Environmental and Molecular Mutagenesis 29, 277–288, doi: 10.1002/(sici)1098-2280(1997)29:3<277::aid-em8>3.0.co;2-9 (1997). 9142171

[b25] Ralph S. & Petras M. Genotoxicity monitoring of small bodies of water using two species of tadpoles and the alkaline single cell gel (comet) assay. Environmental and Molecular Mutagenesis 29, 418–430, doi: 10.1002/(sici)1098-2280(1997)29:4<418::aid-em11>3.0.co;2-h (1997). 9212794

[b26] Ortiz‐Santaliestra M. E., Marco A., Fernández M. J. & Lizana M. Influence of developmental stage on sensitivity to ammonium nitrate of aquatic stages of amphibians. Environmental Toxicology and Chemistry 25, 105–111 (2006). 16494230 10.1897/05-023r.1

[b27] Rohr J. R. . Lethal and sublethal effects of atrazine, carbaryl, endosulfan, and octylphenol on the streamside salamander (*Ambystoma barbour*i). Environmental Toxicology and Chemistry 22, 2385–2392, doi: 10.1897/02-528 (2003). 14552003

[b28] Orton F. & Routledge E. Agricultural intensity in ovo affects growth, metamorphic development and sexual differentiation in the Common toad (*Bufo bufo*). Ecotoxicology 20, 901–911, doi: 10.1007/s10646-011-0658-5 (2011). 21448622

[b29] Bridges C. M. Long-term effects of pesticide exposure at various life stages of the Southern Leopard frog (*Rana sphenocephala*). Archives of Environmental Contamination and Toxicology 39, 91–96, doi: 10.1007/s002440010084 (2000). 10790507

[b30] Malins D. C. . Biomarkers signal contaminant effects on the organs of English sole (*Parophrys vetulus*) from Puget Sound. Environmental Health Perspectives 114, 823–829, doi: 10.1289/ehp.8544 (2006). 16759979 PMC1480518

[b31] Obinaju B. E., Alaoma A. & Martin F. L. Novel sensor technologies towards environmental health monitoring in urban environments: A case study in the Niger Delta (Nigeria). Environmental Pollution 192, 222–231, doi: http://dx.doi.org/10.1016/j.envpol.2014.02.004 (2014). 24602761 10.1016/j.envpol.2014.02.004

[b32] Cakmak G., Togan I. & Severcan F. 17*β*-Estradiol induced compositional, structural and functional changes in rainbow trout liver, revealed by FT-IR spectroscopy: a comparative study with nonylphenol. Aquatic Toxicology 77, 53–63 (2006). 16325934 10.1016/j.aquatox.2005.10.015

[b33] Palaniappan P. L. R. M. & Vijayasundaram V. Fourier transform infrared study of protein secondary structural changes in the muscle of *Labeo rohita* due to arsenic intoxication. Food and Chemical Toxicology 46, 3534–3539, doi: 10.1016/j.fct.2008.09.001 (2008). 18817838

[b34] Llabjani V. . Alterations in the infrared spectral signature of avian feathers reflect potential chemical exposure: A pilot study comparing two sites in Pakistan. Environment International 48, 39–46 (2012). 22832188 10.1016/j.envint.2012.06.019

[b35] Strong R. J. . Biospectroscopy reveals the effect of varying water quality on tadpole tissues of the common frog (*Rana temporaria*). Environmental Pollution 213, 322–337, http://dx.doi.org/10.1016/j.envpol.2016.02.025 (2016). 26925755 10.1016/j.envpol.2016.02.025

[b36] Movasaghi Z., Rehman S. & ur Rehman D. I. Fourier transform infrared (FTIR) spectroscopy of biological tissues. Applied Spectroscopy Reviews 43, 134–179 (2008).

[b37] Trevisan J., Angelov P. P., Carmichael P. L., Scott A. D. & Martin F. L. Extracting biological information with computational analysis of Fourier-transform infrared (FTIR) biospectroscopy datasets: current practices to future perspectives. Analyst 137, 3202–3215, doi: 10.1039/c2an16300d (2012). 22627698

[b38] Ellis D. I. & Goodacre R. Metabolic fingerprinting in disease diagnosis: biomedical applications of infrared and Raman spectroscopy. Analyst 131, 875–885 (2006). 17028718 10.1039/b602376m

[b39] Gómez-Carracedo M. P., Fernández-Varela R., Ballabio D. & Andrade J. M. Screening oil spills by mid-IR spectroscopy and supervised pattern recognition techniques. Chemometrics and Intelligent Laboratory Systems 114, 132–142, doi: http://dx.doi.org/10.1016/j.chemolab.2012.03.013 (2012).

[b40] Gosner K. L. A simplified table for staging anuran embryos and larvae with notes on identification. Herpetologica 16, 183–190 (1960).

[b41] Hands J. . Investigating the rapid diagnosis of gliomas from serum samples using infrared spectroscopy and cytokine and angiogenesis factors. Analytical and Bioanalytical Chemistry 405, 7347–7355, doi: 10.1007/s00216-013-7163-z (2013). 23831829

[b42] Gajjar K. . Fourier-transform infrared spectroscopy coupled with a classification machine for the analysis of blood plasma or serum: a novel diagnostic approach for ovarian cancer. Analyst 138, 3917–3926, doi: 10.1039/c3an36654e (2013). 23325355

[b43] Beebee T. J. C. Amphibian Conservation in Britain: A 40-Year History. Journal of Herpetology 48, 2–12 (2014).

[b44] Dornelles M. F. & Oliveira G. T. Effect of atrazine, glyphosate and quinclorac on biochemical parameters, lipid peroxidation and survival in bullfrog tadpoles (*Lithobates catesbeianus*). Archives of Environmental Contamination and Toxicology 66, 415–429 (2014). 24276472 10.1007/s00244-013-9967-4

[b45] Melvin S. D. . Effects of naphthenic acid exposure on development and liver metabolic processes in anuran tadpoles. Environmental Pollution 177, 22–27, doi: 10.1016/j.envpol.2013.02.003 (2013). 23466728

[b46] Zaya R. M., Amini Z., Whitaker A. S., Kohler S. L. & Ide C. F. Atrazine exposure affects growth, body condition and liver health in *Xenopus laevis* tadpoles. Aquatic Toxicology 104, 243–253, doi: 10.1016/j.aquatox.2011.04.021 (2011). 21635867

[b47] Ezemonye L. I. N. & Tongo I. Lethal and sublethal effects of atrazine to amphibian larvae. Jordan Journal of Biological Science 2, 29–36 (2009).

[b48] Ezemonye L. I. N. & Ilechie I. Acute and chronic effects of organophosphate pesticides (Basudin) to amphibian tadpoles (*Ptychadena bibroni*). African Journal of Biotechnology 6 (2007).

[b49] Gendron A. D., Bishop C. A., Fortin R. & Hontela A. *In vivo* testing of the functional integrity of the corticosterone‐producing axis in mudpuppy (amphibia) exposed to chlorinated hydrocarbons in the wild. Environmental Toxicology and Chemistry 16, 1694–1706 (1997).

[b50] Cakmak G., Togan I., Uğuz C. & Severcan F. FT-IR spectroscopic analysis of rainbow trout liver exposed to nonylphenol. Applied Spectroscopy 57, 835–841 (2003). 14658663 10.1366/000370203322102933

[b51] Audo M. C. . Food deprivation during different periods of tadpole (*Hyla chrysoscelis*) ontogeny affects metamorphic performance differently. Oecologia 103, 518–522 (1995). 10.1007/BF00328691 28307001

[b52] Beebee T. J. C. & Richard G. Amphibians and reptiles. (Collins: London (UK), 2000).

[b53] Alvarez D. & Nicieza A. G. Effects of temperature and food quality on anuran larval growth and metamorphosis. Functional Ecology 16, 640–648, doi: 10.1046/j.1365-2435.2002.00658.x (2002).

[b54] Kupferberg S. J. The role of larval diet in anuran metamorphosis. American Zoologist 37, 146−159 (1997).

[b55] Lewis P. D. . Evaluation of FTIR spectroscopy as a diagnostic tool for lung cancer using sputum. BMC cancer 10, 1 (2010). 21092279 10.1186/1471-2407-10-640 PMC3000851

[b56] Bhargava R., Fernandez D. C., Hewitt S. M. & Levin I. W. High throughput assessment of cells and tissues: Bayesian classification of spectral metrics from infrared vibrational spectroscopic imaging data. Biochimica et Biophysica Acta (BBA) - Biomembranes 1758, 830–845, doi: http://dx.doi.org/10.1016/j.bbamem.2006.05.007 (2006). 10.1016/j.bbamem.2006.05.007 16822477

[b57] Wood B. R. . FTIR microspectroscopic study of cell types and potential confounding variables in screening for cervical malignancies. Biospectroscopy 4, 75–91 (1998). 9557903 10.1002/(SICI)1520-6343(1998)4:2%3C75::AID-BSPY1%3E3.0.CO;2-R

[b58] Theophilou G. . A biospectroscopic analysis of human prostate tissue obtained from different time periods points to a trans-generational alteration in spectral phenotype. Scientific Reports 5, 13465 (2015). 26310632 10.1038/srep13465 PMC4550877

[b59] Neveu A. Incidence of climate on common frog breeding: Long-term and short-term changes. Acta Oecologica 35, 671–678 (2009).

[b60] Carroll E. A., Sparks T. H., Collinson N. & Beebee T. J. C. Influence of temperature on the spatial distribution of first spawning dates of the common frog (*Rana temporaria*) in the UK. Global Change Biology 15, 467–473 (2009).

[b61] Seymour R. S., Roberts J. D., Mitchell N. J. & Blaylock A. J. Influence of environmental oxygen on development and hatching of aquatic eggs of the Australian frog, *Crinia georgiana*. Physiological and Biochemical Zoology 73, 501–507, doi: 10.1086/317739 (2000). 11009404

[b62] Loman J. Microevolution and maternal effects on tadpole *Rana temporaria* growth and development rate. Journal of Zoology 257, 93–99 (2002).

[b63] Taylor S. E. . Infrared spectroscopy with multivariate analysis to interrogate endometrial tissue: a novel and objective diagnostic approach. British Journal of Cancer 104, 790–797, doi: 10.1038/sj.bjc.6606094 (2011). 21326237 PMC3048205

[b64] Maher J. R., Matthews T. E., Reid A. K., Katz D. F. & Wax A. Sensitivity of coded aperture Raman spectroscopy to analytes beneath turbid biological tissue and tissue-simulating phantoms. Journal of Biomedical Optics 19, 117001–117001 (2014). 25371979 10.1117/1.JBO.19.11.117001 PMC4221093

[b65] Martínez A. M. & Kak A. C. Pca versus lda. Pattern Analysis and Machine Intelligence, IEEE Transactions on 23, 228–233 (2001).

[b66] Owens G. L. . Vibrational biospectroscopy coupled with multivariate analysis extracts potentially diagnostic features in blood plasma/serum of ovarian cancer patients. Journal of Biophotonics 7, 200–209 (2014). 24259229 10.1002/jbio.201300157

[b67] Martin F. L. . Distinguishing cell types or populations based on the computational analysis of their infrared spectra. Nature Protocols 5, 1748–1760, doi: 10.1038/nprot.2010.133 (2010). 21030951

[b68] Trevisan J., Angelov P. P., Scott A. D., Carmichael P. L. & Martin F. L. IRootLab: a free and open-source MATLAB toolbox for vibrational biospectroscopy data analysis. Bioinformatics 29, 1095–1097 (2013). 23422340 10.1093/bioinformatics/btt084

[b69] Podrabsky J. E., Carpenter J. F. & Hand S. C. Survival of water stress in annual fish embryos: dehydration avoidance and egg envelope amyloid fibers. American Journal of Physiology - Regulatory, Integrative and Comparative Physiology 280, R123–R131 (2001). 10.1152/ajpregu.2001.280.1.R123 11124142

[b70] Naumann D. FT-infrared and FT-Raman spectroscopy in biomedical research. Applied Spectroscopy Reviews 36, 239–298 (2001).

[b71] Bellisola G. & Sorio C. Infrared spectroscopy and microscopy in cancer research and diagnosis. Am. J. Cancer Res. 2, 1–21 (2012). 22206042 PMC3236568

[b72] Chu H.-L., Liu T.-Y. & Lin S.-Y. Effect of cyanide concentrations on the secondary structures of protein in the crude homogenates of the fish gill tissue. Aquatic Toxicology 55, 171–176 (2001). 11595307 10.1016/s0166-445x(01)00177-1

