# Supplementary material for: Subtle effects of environmental stress observed in the early life stages of the Common frog, Rana temporaria
Source: Sci Rep. 2017 Mar 20;7:44438. doi: 10.1038/srep44438 (PMC5357840; doi:10.1038/srep44438)
Supplement: Supplementary Information [file srep44438-s1.pdf]

Electronic Supplementary Information

Subtle effects of environmental stress observed in the early life stages of the Common frog, Rana temporaria

Rebecca Strong[1], Francis L. Martin[2*], Kevin C. Jones[1], Richard F. Shore[3] and Crispin J. Halsall[1*]

[1]*Lancaster Environment Centre, Lancaster University, Bailrigg, Lancaster LA1 4YQ, UK*

[2]*School of Pharmacy and Biomedical Sciences, University of Central Lancashire (UCLan), Preston PR1 2HE, UK*

[3]*Centre for Ecology and Hydrology, Lancaster University, Bailrigg, Lancaster LA1 4YQ, UK*

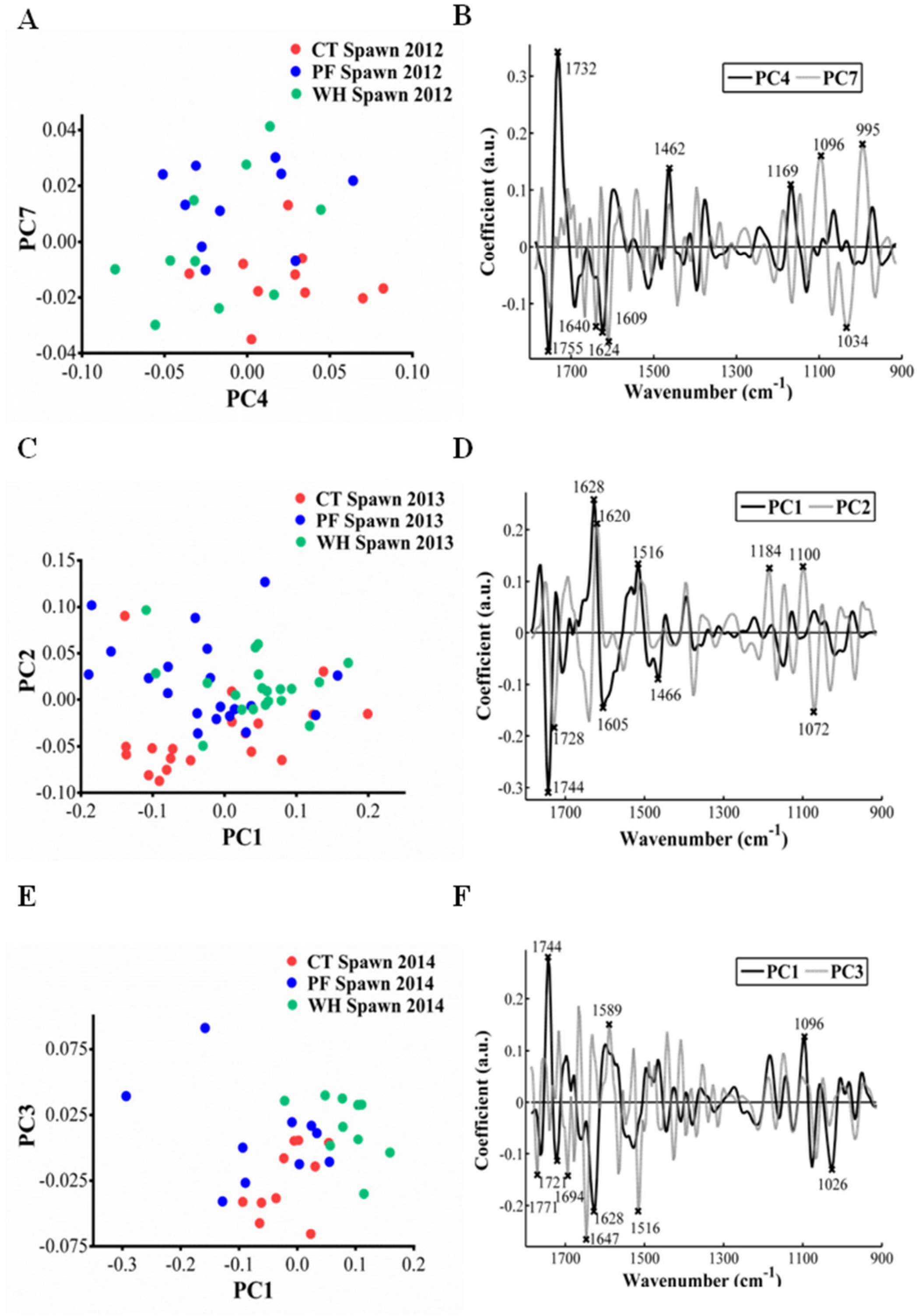

Supplementary Figure 1. *Rana temporaria* spawn collected from three different ponds separated into year groups and analysed with PCA following interrogation with ATR-FTIR spectroscopy. A. Scores and B. Loadings plots of spawn collected in 2012; C. Scores and D. Loadings plots of spawn collected in 2013 and E. Scores and F. Loadings plots of spawn collected in 2014. Ponds are: CT: a rural agricultural pond with minimal pesticide input; PF: an urban pond impacted by wastewater and landfill run-off and WH: an agricultural pond known to be impacted by pesticides.

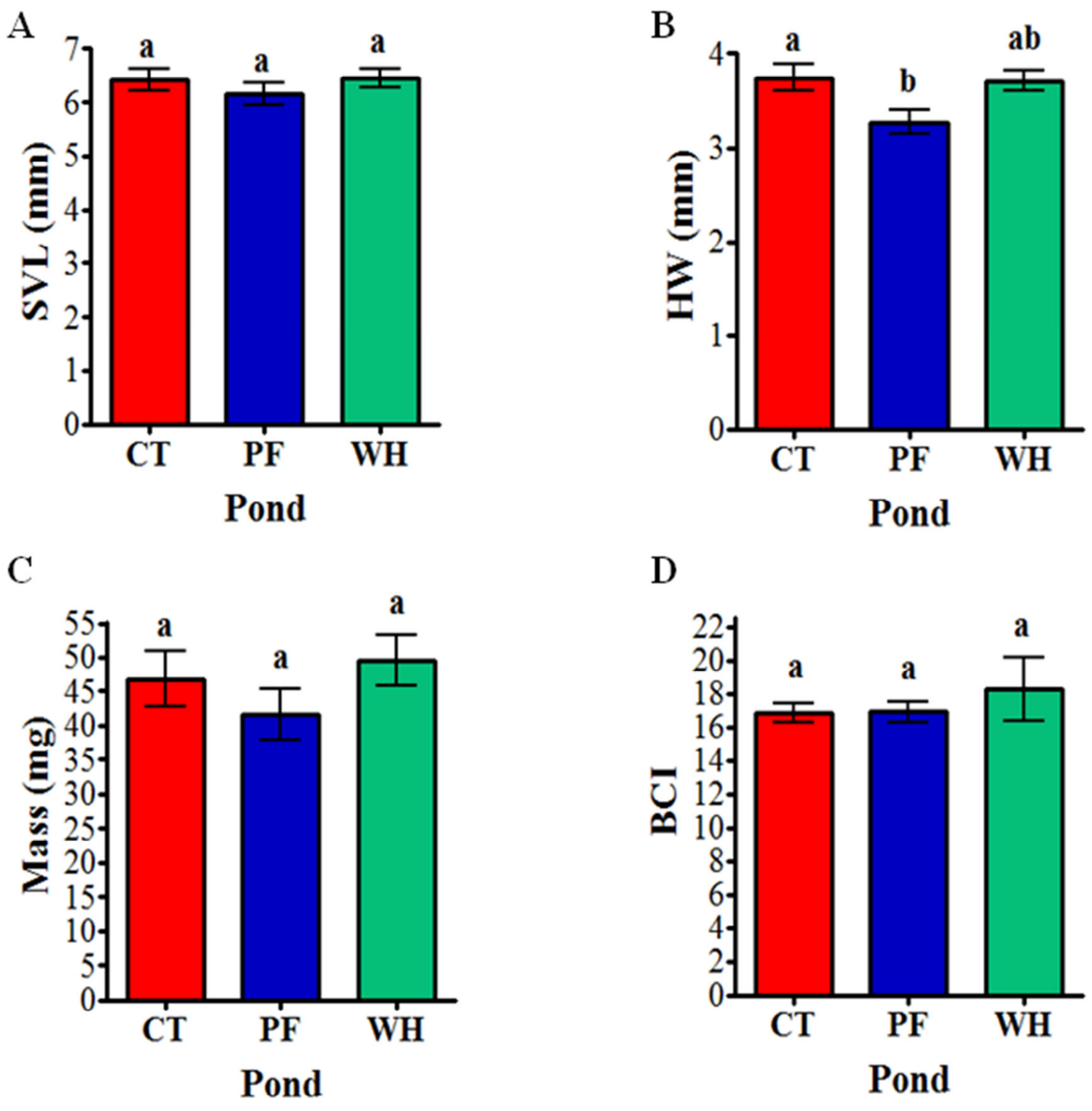

Supplementary Figure 2. Comparison of body size parameters of *Rana temporaria* tadpoles collected over a three year period (2012-2014). Comparisons were made between tadpoles from CT: a rural agricultural pond with minimal pesticide input; PF: an urban pond impacted by wastewater and landfill run-off and WH: an agricultural pond known to be impacted by pesticides. Measurements are (A) snout-vent-length (SVL), (B) head width (HW) (C) body mass, and (D) body condition index (BCI). One-way ANOVA followed by Tukey's multiple comparison tests were used to compare each body size parameter between ponds. Different letters denote a significant difference (P <0.05).

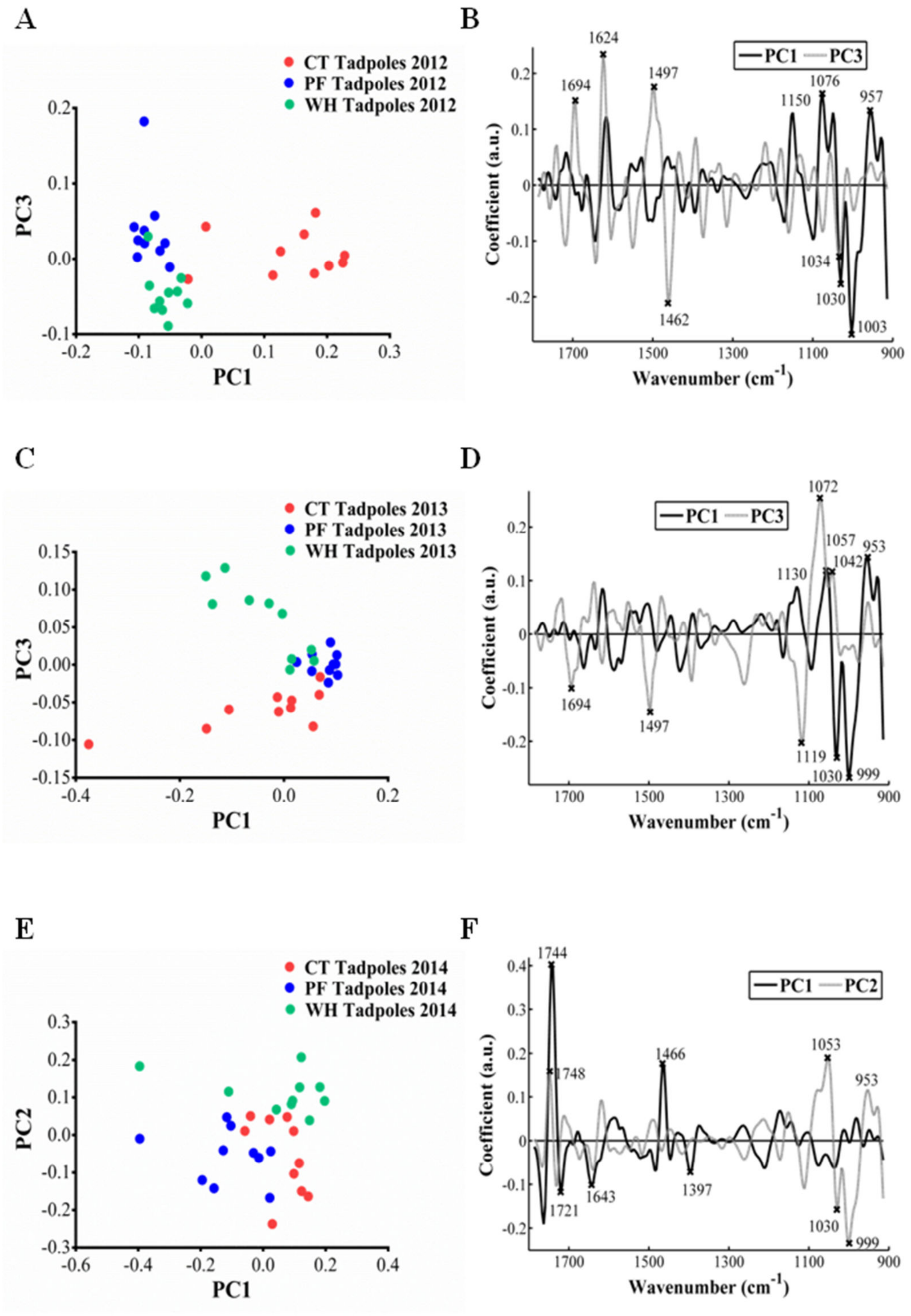

Supplementary Figure 3. *Rana temporaria* tadpoles collected from three different ponds separated into year groups and analysed with PCA following interrogation with ATR-FTIR spectroscopy. A. Scores and B. Loadings plots of tadpoles collected in 2012; C. Scores and D. Loadings plots of tadpoles collected in 2013 and E. Scores and F. Loadings plots of tadpoles collected in 2014. Ponds are: CT: a rural agricultural pond with minimal pesticide input; PF: an urban pond impacted by wastewater and landfill run-off and WH: an agricultural pond known to be impacted by pesticides.

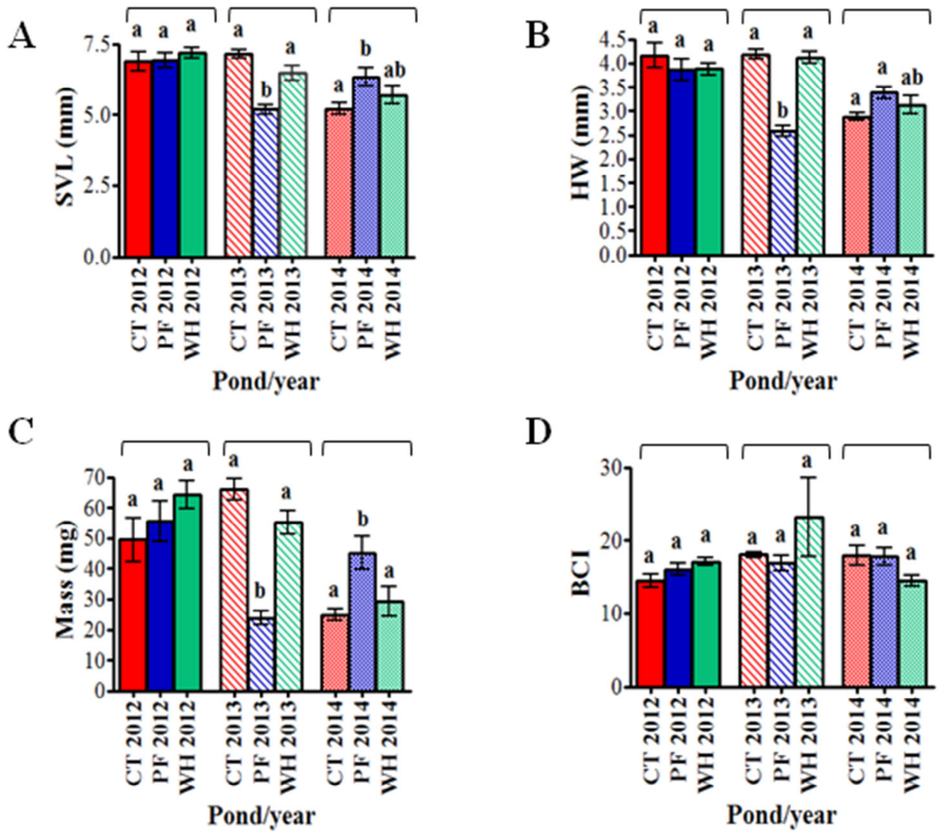

Supplementary Figure 4. Comparison of body size parameters of *Rana temporaria* tadpoles collected from ponds with differing water quality for the years 2012, 2013 and 2014. Comparisons of body size measures were made between ponds looking at each year separately using one-way ANOVA and Tukey's multiple comparison tests. Different letters denote a significant difference where $P < 0.05$. Ponds are CT: a rural agricultural pond with minimal pesticide input; PF: an urban pond impacted by wastewater and landfill run-off and WH: an agricultural pond known to be impacted by pesticides. Measurements are: **(A)** snout-vent-length (SVL), **(B)** head width (HW), **(C)** body mass, and **(D)** body condition index (BCI).

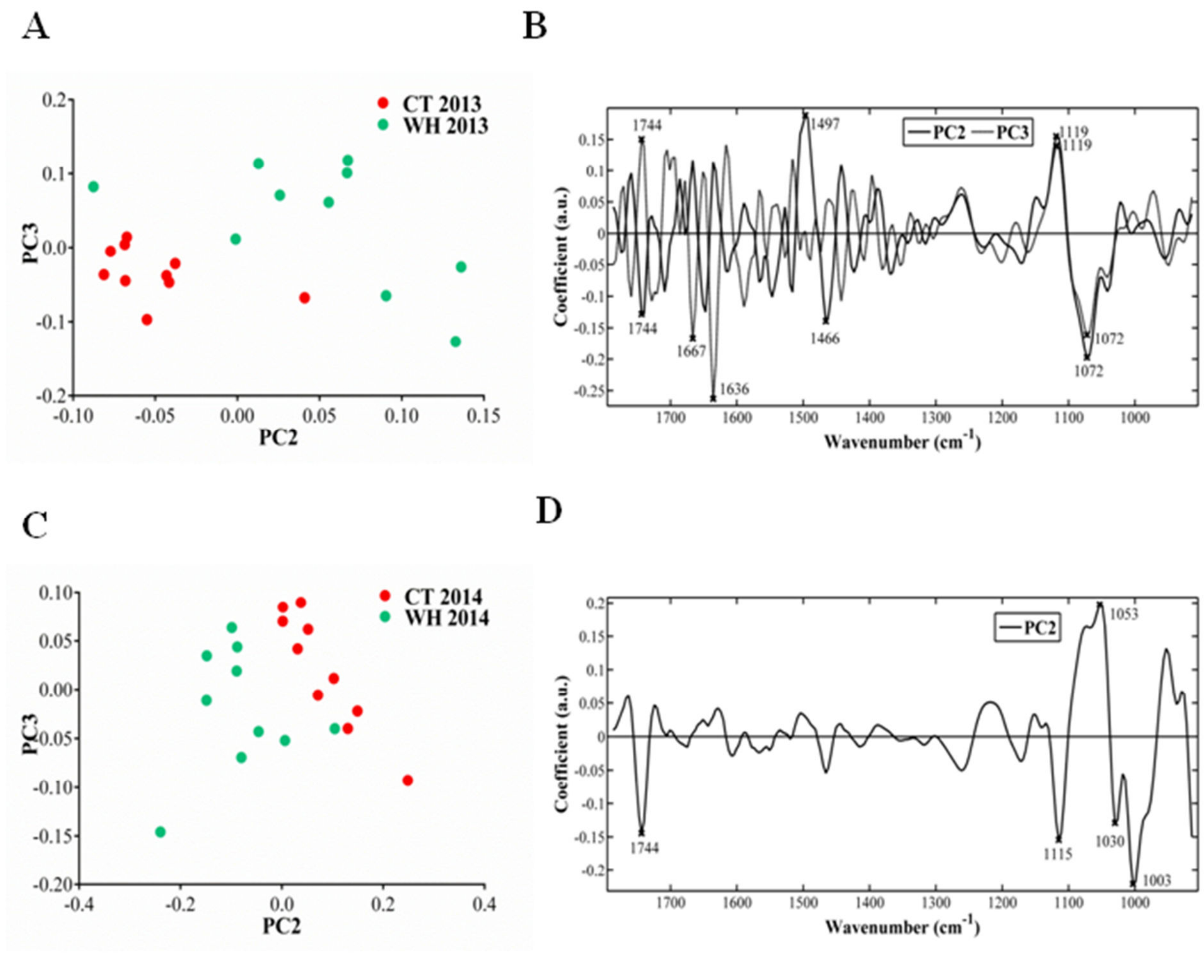

Supplementary Figure 5. *Rana temporaria* tadpoles collected from two different ponds separated into year groups and analysed with PCA following interrogation with ATR-FTIR spectroscopy. **A.** Scores and **B.** Loadings plots of tadpoles collected in 2013; **C.** Scores and **D.** Loadings plots of tadpoles collected in 2014. Ponds are: CT: a rural agricultural pond with minimal pesticide input and WH: an agricultural pond known to be impacted by pesticides. Tadpoles from PF were excluded from analysis due to body size differences.

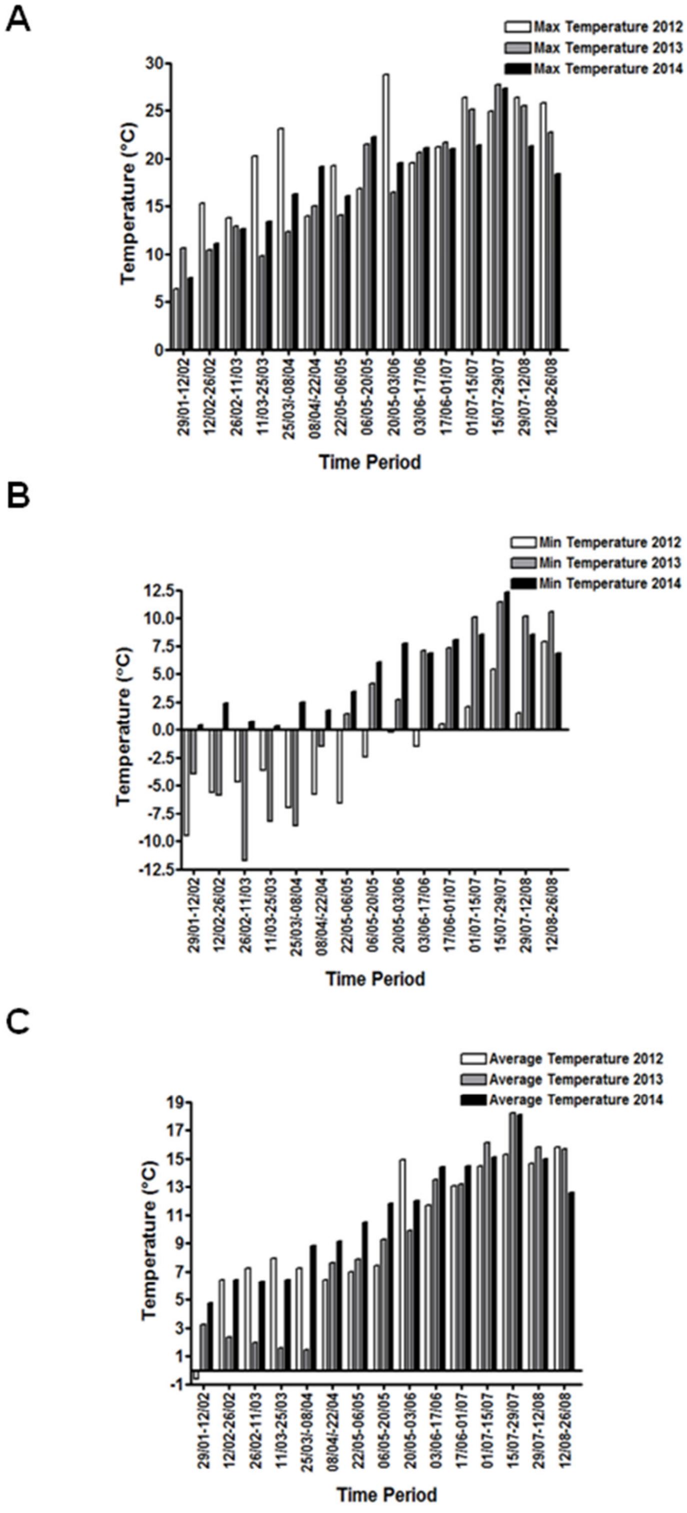

Supplementary Figure 6. Maximum (A), minimum (B) and average (C) air temperatures collected from Hazelrigg Weather station at Lancaster University over two week time periods from a month before the beginning of the common frog breeding season to after metamorphosis of tadpoles.

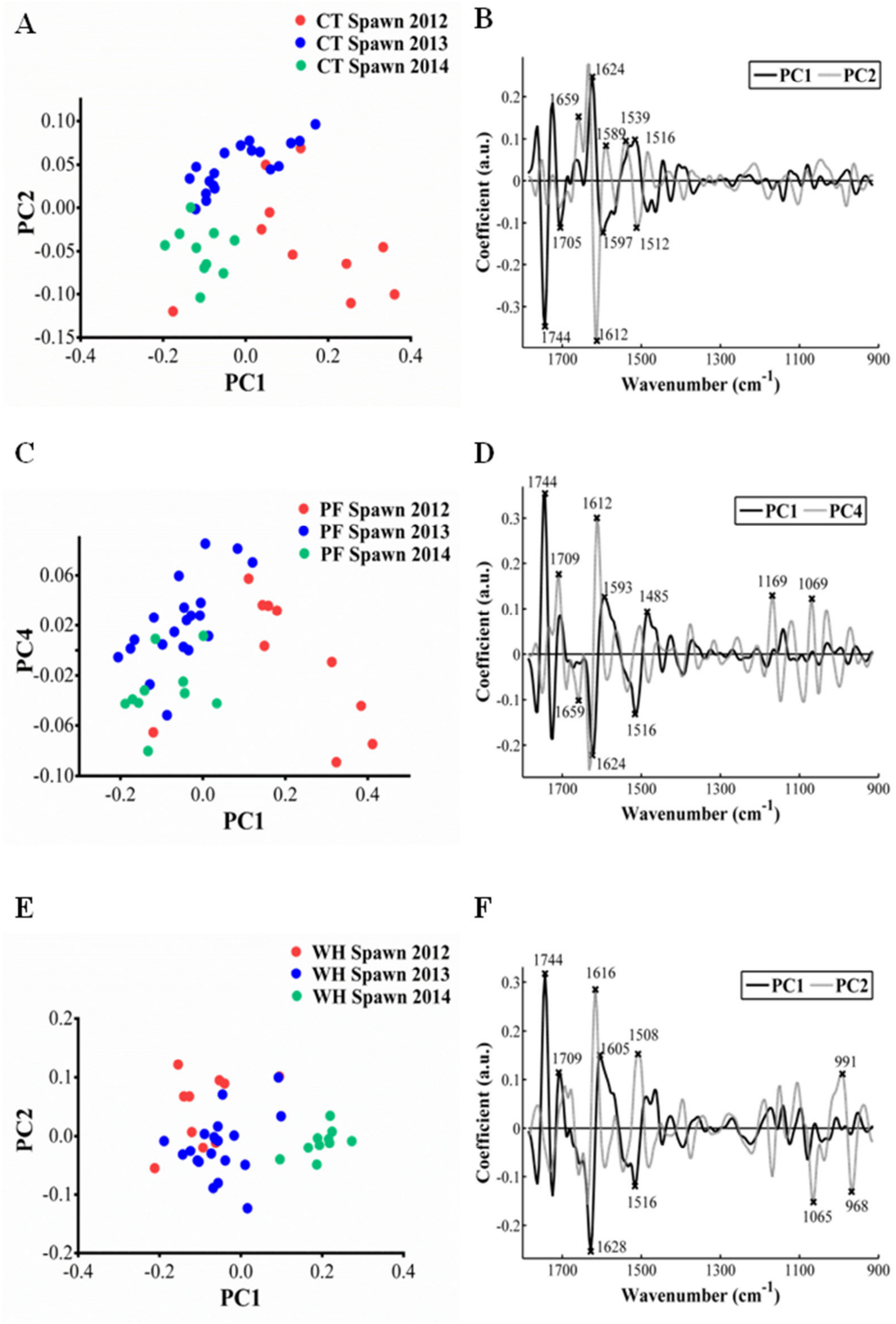

Supplementary Figure 7. *Rana temporaria* spawn collected from three different ponds separated into year groups and analysed with PCA following interrogation with ATR-FTIR spectroscopy. A. Scores and B. Loadings plots of spawn collected in 2012; C. Scores and D. Loadings plots of spawn collected in 2013 and E. Scores and F. Loadings plots of spawn collected in 2014. Ponds are: CT: a rural agricultural pond with minimal pesticide input; PF: an urban pond impacted by wastewater and landfill run-off and WH: an agricultural pond known to be impacted by pesticides.

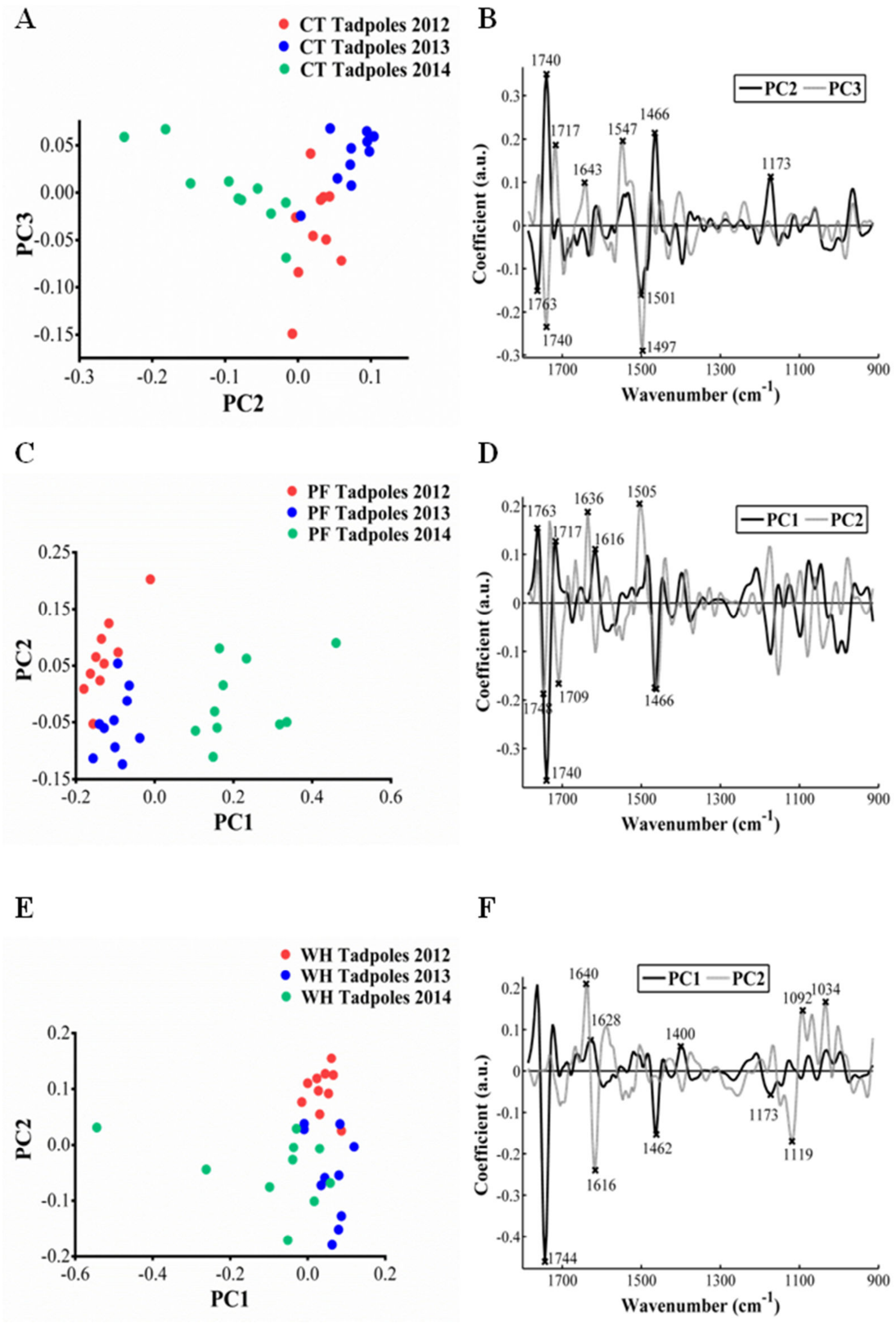

Supplementary Figure 8. *Rana temporaria* tadpoles collected from three different ponds separated into year groups and analysed with PCA following interrogation with ATR-FTIR spectroscopy. **A.** Scores and **B.** Loadings plots of tadpoles collected in 2012; **C.** Scores and **D.** Loadings plots of tadpoles collected in 2013 and **E.** Scores and **F.** Loadings plots of tadpoles collected in 2014. Ponds are: CT: a rural agricultural pond with minimal pesticide input; PF: an urban pond impacted by wastewater and landfill run-off and WH: an agricultural pond known to be impacted by pesticides.

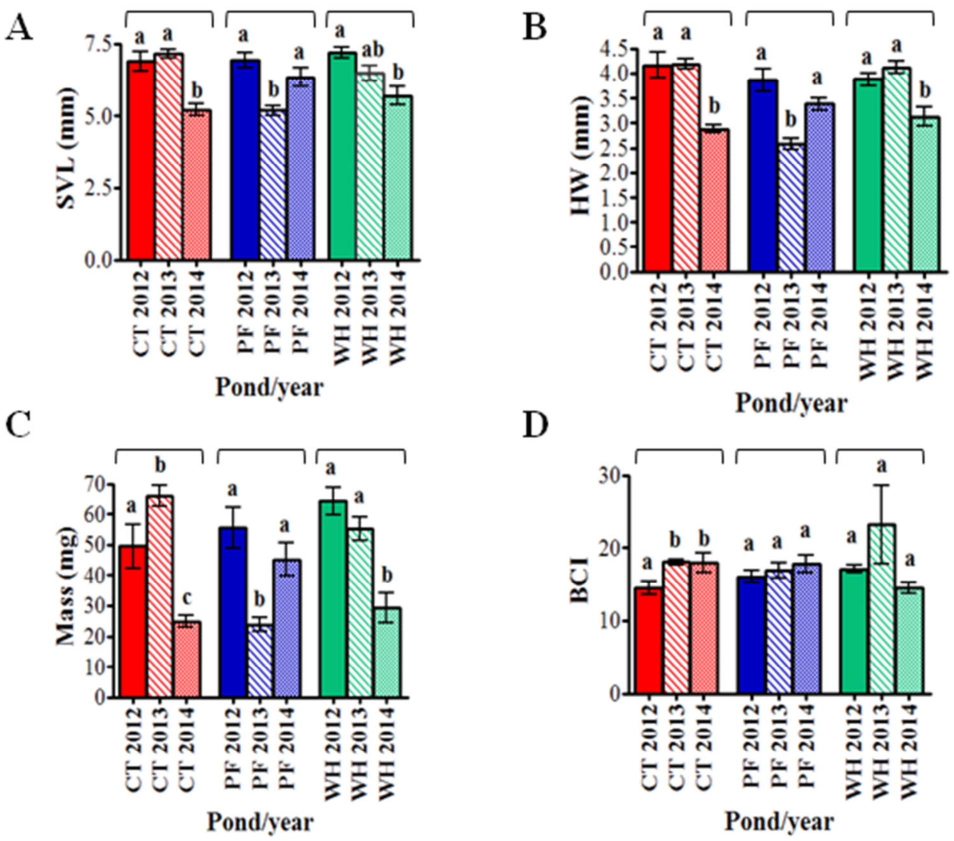

Supplementary Figure 9. Comparison of body size parameters of *Rana temporaria* tadpoles collected from the same ponds over a three year period (2012-2014). Comparisons were made between body size parameters within each pond between years using one-way ANOVA and Tukey's multiple comparison tests. Different letters denote a significant difference where $P < 0.05$. Ponds are CT: a rural agricultural pond with minimal pesticide input; PF: an urban pond impacted by wastewater and landfill run-off and WH: an agricultural pond known to be impacted by pesticides. Measurements are: **(A)** snout-vent-length (SVL), **(B)** head width (HW), **(C)** body mass, and **(D)** body condition index (BCI).

Supplementary Table 1. Distinguishing wavenumbers and proposed assignments obtained from analysis of *Rana temporaria* spawn with ATR-FTIR spectroscopy following analysis with PCA. The five largest loadings values for the principal components which best separated the data following one-way ANOVA are shown. Comparisons were made between ponds: CT: a rural agricultural pond with no pesticide input; WH: an agricultural pond known to be impacted by pesticides and PF: an urban pond impacted by wastewater and landfill run-off.

Comparison	Wavenumber (cm^{-1})	Tentative Assignment [¥]	Differences [≠]
**Spawn 2012**			
PC4	1755	Lipid fatty acids	CT [a]
	1732	Fatty acid esters	PF [ab]
	1462	CH_2 acyl chain of lipid	WH [b]
	1624	Amide I, β-sheet	
	1169	Asymmetric stretching CO-O-C	
PC6	1640	Amide I protein	CT [a]
	1609	Adenine vibration DNA	PF [b]
	1096	Phosphate II stretching (asymmetric) in RNA	WH [ab]
	1034	Collagen	
	995	Ring breathing	
**Spawn 2013**			
PC1	1744	C=O stretching mode of lipids	CT [ab]
	1628	Amide I	PF [a]
	1605	Asymmetric stretch polysaccharides and pectin	WH [b]
	1516	Amide II	
	1466	CH_2 scissoring mode of the acyl chain of lipid	
PC2	1728	C=O band	CT [a]
	1620	Peak of nucleic acids due to the base carbonyl stretching and ring breathing mode	PF [b]<br>WH [b]
	1184	Amide III	
	1100	Stretching PO_2^- symmetric (phosphate II)	
	1072	Nucleic acid band	
**Spawn 2014**			
PC1	1744	C=O stretching mode of lipids	CT [a]
	1721	C=O stretching	PF [a]
	1628	Amide I	WH [b]
	1096	Phosphate II stretching (asymmetric) in RNA	
	1026	Glycogen	
PC3	1771	Fatty acid esters	CT [a]
	1694	Amide I vibration	PF [b]
	1647	Amide I	WH [b]
	1589	Ring C-C stretch of phenyl	
	1516	Amide II	

¥ 1,6,7

[≠] Different letters denote a significant difference at the P <0.05 level following one-way ANOVA and Tukey's multiple comparison tests.

Supplementary Table 2. Distinguishing wavenumbers and proposed assignments obtained from analysis of *Rana temporaria* tadpoles with ATR-FTIR spectroscopy following analysis with PCA. The five largest loadings values for the principal components which best separated the data following one-way ANOVA are shown. Comparisons were made between sites for each year (2012, 2013, 2014) as follows: CT: a rural agricultural pond with no pesticide input; WH: an agricultural pond known to be impacted by pesticides and PF: an urban pond impacted by wastewater and landfill run-off.

Comparison	Wavenumber (cm^{-1})	Tentative Assignment [¥]	Differences [≠]
**Tadpole 2012**			
PC1	1150	C-O stretching of carbohydrates	CT [a]
	1076	Symmetric phosphate stretching	PF [b]
	1030	Glycogen	WH [b]
	1003	Sugar phosphate chain vibrations in nucleic acids	
	957	Symmetric stretching vibration of phosphate	
PC3	1694	Amide I vibration	CT [a]
	1624	Amide I, β-sheet	PF [a]
	1497	C=C, deformation C-H	WH [b]
	1462	CH_2 acyl chain of lipid	
	1034	Glycogen/collagen	
**Tadpole 2013**			
PC1	1130	Polysaccharides	CT [a]
	1057	Stretching C-O deoxyribose	PF [b]
	1030	Glycogen	WH [a]
	999	Ring stretching vibration	
	953	Phosphodiester region	
PC3	1694	Amide I vibration	CT [a]
	1497	C=C, deformation C-H	PF [b]
	1119	Symmetric stretching P-O-C	WH [c]
	1072	Nucleic acid band	
	1042	Glycogen	
**Tadpole 2014**			
PC1	1744	C=O stretching mode of lipids	CT [a]
	1721	C=O	PF [b]
	1643	Amide I band (from C=O stretching)	WH [a]
	1466	CH2 scissoring mode of the acyl chain of lipid	
	1397	CH_3 bending/deformation	
PC2	1748	Lipids/fatty acids (C=C)	CT [a]
	1053	C-O stretching carbohydrates	PF [a]
	1030	Glycogen	WH [b]
	999	Ring stretching vibration	
	953	Phosphodiester region	

¥ 1,8-10

≠ Different letters denote a significant difference at the P <0.05 level following one-way ANOVA and Tukey's multiple comparison tests.

Supplementary Table 3. Distinguishing wavenumbers and proposed assignments obtained from analysis of *Rana temporaria* tadpoles with ATR-FTIR spectroscopy following analysis with PCA. The five largest loadings values for the principal components which best separated the data following one-way ANOVA are shown. Tadpoles from PF were removed from analysis due to body size differences between tadpoles from this site and those from CT and WH; the loadings therefore represent the areas of the spectrum attributable to differences between CT and WH only.

Comparison	Wavenumber (cm^{-1})	Tentative Assignment ¥	Differences ≠
**Tadpole 2013**			
PC2	1744	C=O stretching mode of lipids	CT [a]
	1497	C=C, deformation C-H	WH [b]
	1466	CH_2 scissoring mode of the acyl chain of lipid	
	1119	Symmetric stretching P-O-C	
	1072	Nucleic acid band	
PC3	1744	C=O stretching mode of lipids	CT [a]
	1667	Amide I	WH [b]
	1636	Amide I	
	1119	Symmetric stretching P-O-C	
	1072	Nucleic acid band	
**Tadpole 2014**			
PC2	1744	C=O stretching mode of lipids	CT [a]
	1115	Symmetric stretching P-O-C	WH [b]
	1053	C-O stretching carbohydrates	
	1030	Glycogen	
	1003	Sugar phosphate chain vibrations in nucleic acids	

¥ (Cakmak et al., 2006; Chu et al., 2001; Movasaghi et al., 2008; Palaniappan and Vijayasundaram, 2008)

≠ Different letters denote a significant difference at the $P < 0.05$ level following analysis with two-sample *t*-tests.

Supplementary Table 4. Dates spawn samples of *Rana temporaria* were collected from the three ponds studied over a three year period. Ponds are CT: a rural agricultural pond with minimal pesticide impact, PF: an urban pond exposed to wastewater/landfill run-off and WH: an agricultural pond impacted by pesticides.

Pond	Year	Date Collected
CT	2012	07/03/2012
CT	2013	11/04/2013
CT	2014	07/03/2014
PF	2012	16/03/2013
PF	2013	27/03/2013
PF	2014	12/03/2014
WH	2012	07/03/2012
WH	2013	11/04/2013
WH	2014	07/03/2014

Supplementary Table 5. Full details of tadpoles collected from each pond over the period 2012-2014. Ponds are CT: a rural agricultural pond with minimal pesticide impact, PF: an urban pond exposed to wastewater/landfill run-off and WH: an agricultural pond impacted by pesticides. SVL = snout-vent-length, HW = head width, GS = Gosner Stage.

Pond	Year	Collection Date	Number	SVL (mm)	HW (mm)	Mass (mg)	BCI	GS
CT	2012	18/04/2012	1	7.22	4.64	59.00	15.68	25
CT	2012	18/04/2012	2	6.87	3.82	60.90	18.78	25
CT	2012	18/04/2012	3	8.03	4.83	84.70	16.36	25
CT	2012	18/04/2012	4	7.00	4.34	52.00	15.16	27
CT	2012	18/04/2012	5	5.86	3.42	25.00	12.42	25
CT	2012	18/04/2012	6	5.74	3.52	33.00	17.45	25
CT	2012	18/04/2012	7	6.55	3.73	35.00	12.46	25
CT	2012	18/04/2012	8	9.32	6.06	84.00	10.38	25
CT	2012	18/04/2012	9	6.15	3.50	34.70	14.92	28
CT	2012	18/04/2012	10	6.17	3.78	26.00	11.07	25
CT	2013	31/05/2013	1	6.88	3.70	60.90	18.70	25
CT	2013	31/05/2013	2	6.96	4.47	66.40	19.69	26
CT	2013	31/05/2013	3	7.36	4.29	74.20	18.61	26
CT	2013	31/05/2013	4	6.63	3.98	52.30	17.95	26
CT	2013	31/05/2013	5	7.66	4.36	68.40	15.22	25
CT	2013	31/05/2013	6	7.61	4.64	80.60	18.29	26
CT	2013	31/05/2013	7	7.20	4.06	61.50	16.48	26
CT	2013	31/05/2013	8	7.77	4.49	84.70	18.06	27
CT	2013	31/05/2013	9	6.76	3.73	59.60	19.29	27
CT	2013	31/05/2013	10	6.64	4.08	52.70	18.00	25
CT	2014	11/04/2014	1	4.69	2.74	25.00	24.23	25
CT	2014	11/04/2014	2	5.13	2.78	28.00	20.74	25
CT	2014	11/04/2014	3	4.02	2.29	14.00	21.55	25
CT	2014	11/04/2014	4	5.47	2.89	22.00	13.44	25
CT	2014	11/04/2014	5	4.77	3.05	23.00	21.19	25
CT	2014	11/04/2014	6	5.09	2.96	24.00	18.20	25
CT	2014	11/04/2014	7	6.28	3.20	40.00	16.15	25
CT	2014	11/04/2014	8	5.87	3.15	25.00	12.36	25
CT	2014	11/04/2014	9	5.57	2.80	23.00	13.31	25
CT	2014	11/04/2014	10	5.15	2.96	25.00	18.30	25
PF	2012	17/04/2012	1	8.60	5.18	84.00	13.21	26
PF	2012	17/04/2012	2	6.44	3.04	40.00	14.98	25
PF	2012	17/04/2012	3	7.16	4.16	61.50	16.75	25
PF	2012	17/04/2012	4	6.17	2.97	26.00	11.07	25
PF	2012	17/04/2012	5	6.16	3.47	34.70	14.85	25
PF	2012	17/04/2012	6	7.42	4.41	74.20	18.16	25
PF	2012	17/04/2012	7	7.20	3.57	61.50	16.48	26
PF	2012	17/04/2012	8	7.54	4.47	80.60	18.80	26
PF	2012	17/04/2012	9	6.84	3.95	60.90	19.03	26
PF	2012	17/04/2012	10	6.84	3.95	60.90	19.03	25
PF	2013	30/05/2015	1	4.72	2.11	18.80	17.88	25
PF	2013	30/05/2015	2	4.81	2.81	25.20	22.64	25
PF	2013	30/05/2015	3	5.50	2.99	35.20	21.16	25
PF	2013	30/05/2015	4	6.15	2.95	34.70	14.92	26
PF	2013	30/05/2015	5	4.99	2.31	21.60	17.38	26
PF	2013	30/05/2015	6	5.26	2.28	16.40	11.27	25
PF	2013	30/05/2015	7	5.36	2.64	20.40	13.25	25
PF	2013	30/05/2015	8	5.95	2.93	31.10	14.76	25

PF	2013	30/05/2015	9	4.58	2.26	16.20	16.86	25
PF	2013	30/05/2015	10	4.60	2.54	18.20	18.70	25
PF	2014	16/04/2014	1	6.51	3.94	60.00	21.75	26
PF	2014	16/04/2014	2	6.31	3.49	41.00	16.32	25
PF	2014	16/04/2014	3	5.71	3.15	33.00	17.73	25
PF	2014	16/04/2014	4	8.86	4.17	84.00	12.08	25
PF	2014	16/04/2014	5	6.40	3.50	44.00	16.78	26
PF	2014	16/04/2014	6	5.23	3.04	36.00	25.17	25
PF	2014	16/04/2014	7	6.67	3.44	55.00	18.53	25
PF	2014	16/04/2014	8	5.69	2.98	38.00	20.63	25
PF	2014	16/04/2014	9	5.40	2.91	26.00	16.51	25
PF	2014	16/04/2014	10	6.54	3.19	35.00	12.51	25
WH	2012	18/04/2012	1	8.09	4.26	84.70	16.00	28
WH	2012	18/04/2012	2	7.59	4.16	80.60	18.43	28
WH	2012	18/04/2012	3	6.33	3.65	41.00	16.16	26
WH	2012	18/04/2012	4	7.31	4.03	74.20	19.00	27
WH	2012	18/04/2012	5	6.65	3.35	52.70	17.92	27
WH	2012	18/04/2012	6	7.71	4.46	68.40	14.92	26
WH	2012	18/04/2012	7	6.95	3.70	66.40	19.78	28
WH	2012	18/04/2012	8	7.17	3.74	61.50	16.68	27
WH	2012	18/04/2012	9	6.37	3.29	44.00	17.02	26
WH	2012	18/04/2012	10	7.65	4.15	68.40	15.28	26
WH	2013	31/05/2013	1	6.79	4.33	50.20	16.04	27
WH	2013	31/05/2013	2	7.21	4.56	59.00	15.74	26
WH	2013	31/05/2013	3	6.99	3.93	52.00	15.23	27
WH	2013	31/05/2013	4	6.66	3.92	51.40	17.38	26
WH	2013	31/05/2013	5	4.54	4.37	66.90	71.49	26
WH	2013	31/05/2013	6	7.03	4.66	68.00	19.57	28
WH	2013	31/05/2013	7	6.92	4.24	73.60	22.21	27
WH	2013	31/05/2013	8	5.63	3.39	36.00	20.17	28
WH	2013	31/05/2013	9	6.75	4.08	52.40	17.04	26
WH	2013	31/05/2013	10	6.24	3.70	42.00	17.29	26
WH	2014	11/04/2014	1	6.18	3.16	26.00	11.02	25
WH	2014	11/04/2014	2	6.47	3.63	40.00	14.77	25
WH	2014	11/04/2014	3	4.23	2.39	11.00	14.53	25
WH	2014	11/04/2014	4	6.94	3.66	54.00	16.16	25
WH	2014	11/04/2014	5	4.33	2.11	9.00	11.09	25
WH	2014	11/04/2014	6	6.25	3.67	39.00	15.97	25
WH	2014	11/04/2014	7	5.43	3.05	28.00	17.49	25
WH	2014	11/04/2014	8	6.88	3.86	47.00	14.43	25
WH	2014	11/04/2014	9	4.92	2.78	20.00	16.79	25
WH	2014	11/04/2014	10	5.32	2.98	19.00	12.62	25

Supplementary Table 6. Distinguishing wavenumbers and proposed assignments obtained from analysis of *Rana temporaria* spawn with ATR-FTIR spectroscopy following analysis with PCA. The five largest loadings values for the two most discriminating principal components as determined by one-way ANOVA are shown. Comparisons were made between years for each site sampled. Sites are as follows: CT: a rural agricultural pond with no pesticide input; WH: an agricultural pond known to be impacted by pesticides and PF: an urban pond impacted by wastewater and landfill run-off.

Comparison	Wavenumber (cm^{-1})	Tentative Assignment [¥]	Differences [≠]
**CT Spawn**			
PC1	1744	C=O stretching of lipids	2012 [a]
	1705	C=O stretching (bases)	2013 [b]
	1624	Amide I, *β*-sheet	2014 [b]
	1597	C=N, NH_2 adenine	
	1516	Amide II	
PC2	1659	Amide I	2012 [a]
	1612	Amide I	2013 [b]
	1589	Ring C-C stretch of phenyl	2014 [a]
	1539	Amide II	
	1512	Amide II	
**PF Spawn**			
PC1	1744	C=O stretching of lipids	2012 [a]
	1624	Amide I, *β*-sheet	2013 [b]
	1593	Ring C-C stretch of phenyl	2014 [b]
	1516	Amide II	
	1485	C-H deformation	
PC4	1709	C=O stretching (bases)	2012 [ab]
	1659	Amide I	2013 [a]
	1612	Amide I (carbonyl stretching vibrations in side chains of amino acids)	2014 [b]
	1169	C-O bands from glycomaterials and proteins	
	1069	Stretching C-O ribose	
**WH Spawn**			
PC1	1744	C=O stretching of lipids	2012 [a]
	1709	C=O stretching (bases)	2013 [a]
	1628	Amide I (Intramolecular *β*-sheet)	2014 [b]
	1605	Asymmetric stretching polysaccharides	
	1516	Amide II	
PC2	1616	Amide I	2012 [a]
	1508	In-plane CH bending vibration from the phenyl rings	2013 [b]<br>2014 [b]
	1065	C-O stretching of ribose and phosphodiester	
	991	C-O deoxyribose	
	968	Phosphodiester region	

¥ 1,6,7

≠ Different letters denote a significant difference at the *P* <0.05 level following one-way ANOVA and Tukey's multiple comparison tests.

Supplementary Table 7. Distinguishing wavenumbers and proposed assignments obtained from analysis of *Rana temporaria* tadpoles with ATR-FTIR spectroscopy following analysis with PCA. The five largest loadings values for the two most discriminating principal components as determined by one-way ANOVA are shown. Comparisons were made between years for each site sampled. Sites are as follows: CT: a rural agricultural pond with no pesticide input; WH: an agricultural pond known to be impacted by pesticides and PF: an urban pond impacted by wastewater and landfill run-off.

Comparison	Wavenumber (cm^{-1})	Tentative Assignment ¥	Differences ≠
**CT Tadpole**			
PC2	1763	Lipid	2012 [a]
	1740	C=O stretching (lipids)	2013 [a]
	1501	Amide II bending	2014 [b]
	1466	CH_2 scissoring mode of the acyl chain of lipid	
	1173	C-O stretching of protein and carbohydrate	
PC3	1740	C=O stretching (lipids)	2012 [a]
	1717	C=O stretching vibration	2013 [b]
	1643	Amide I	2014 [ab]
	1547	Amide II	
	1497	C=C, deformation C-H	
**PF Tadpole**			
PC1	1763	Fatty acid esters	2012 [a]
	1740	C=O stretching (lipids)	2013 [a]
	1717	C=O stretching vibration	2014 [b]
	1616	Amide I	
	1466	CH_2 scissoring mode of the acyl chain of lipid	
PC2	1748	Lipids/fatty acids (C=C)	2012 [a]
	1709	C=O stretching (bases)	2013 [b]
	1636	Amide I	2014 [b]
	1505	In-plane CH bending vibration from the phenyl rings	
	1466	CH_2 scissoring mode of the acyl chain of lipid	
**WH Tadpole**			
PC1	1744	C=O stretching (lipids)	2012 [a]
	1628	Amide I	2013 [a]
	1462	CH_2 scissoring mode of the acyl chain of lipid	2014 [b]
	1400	Symmetric bending/stretching of methyl groups in proteins	
	1173	C-O stretching of protein and carbohydrate	
PC2	1640	Amide I	2012 [a]
	1616	Amide I	2013 [b]
	1119	Symmetric stretching P-O-C	2014 [b]
	1092	Stretching PO_2^- symmetric	
	1034	Glycogen	

¥ 1,8-10

≠ Different letters denote a significant difference at the $P < 0.05$ level following one-way ANOVA and Tukey's multiple comparison tests.

Supplementary Notes

Supplementary Note 1: Theory behind classification techniques

PCA-LDC Classifier

Classifiers may be generated using PCA first as a data reduction tool so that the data are not over-fitted in the subsequent LDC model; the classification version of LDA [11]. LDC generates *n*-1 linear discriminant functions (2 in this study) which optimally discriminate *n* classes (3 classes per data set in this study). LDC uses these discriminant functions to assign unknown observations to classes. The Mahalanobis distance (the distance between a data point and a multivariate space's overall mean) is used in the classification process, as the group with the smallest distance is the one LDC classifies the observation into [12].

Data were pre-processed as for PCA-LDA , standardised and the number of principal components for input into the classification model was selected as before using the PCA Pareto function in Matlab before input into the classifier. Five-fold cross-validation was implemented in order to prevent the model from being over-fitted during the training phase.

SVM Classifier

SVM is a machine-learning approach, which aims to separate data classes by a hyperplane, which maximises the margin between different classes while giving a low generalisation error [13]. Given the labelled training dataset, the model creates an optimal hyperplane, which then classifies new examples. The points determining the hyperplane are called support vectors [14]. SVMs can be linear, however they can also be useful for data that cannot be separated linearly; in this case the predictors are mapped onto a new higher-dimension space, where they can be separated linearly, which is known as the 'kernel trick' [15]. Although originally designed to solve binary classification problems, they can also be applied to multiclass

problems by creating several binary classifiers and combining them. The most common approaches for multiclass datasets are “one-against-one”, which creates a separate SVM for each class or “one-against-all”, which creates a separate SVM for each pair of classes [13].

In this study, the SVM was set up using a “one-against-one” approach using the LibSVM library [16] in Matlab (software available at http://www.csie.ntu.edu.tw/~cjlin/libsvm), as there were three classes in each data set. Prior to the application of the SVM classifier, the data were pre-processed as before and then each variable was linearly scaled to the [0, 1] range. Scaling avoids variables in larger numeric ranges from dominating those in smaller ranges and prevents numerical difficulties during the calculations [17]. A radial basis kernel function (RBF) was employed and the optimum penalty parameter, C, and the kernel function parameter, γ, were found using the grid search algorithm. This approach identifies the C, γ pairs with the best cross-validation accuracy [18]. Five-fold cross-validation was conducted in order to prevent over-fitting during the training process (as in the PCA-LDC classifier for comparison).

Supplementary Note 2: Analysis of peak heights

Detailed quantification of differences between samples at specific wavenumbers was also implemented using absorbance values from the second derivatives. This is a technique which has previously been implement using data from IR spectroscopy studies. The second derivative has its maximum value at the same value as the underlying absorbance peak but in the negative direction. Spectra were pre-processed as before (cut in the 1800-900 cm^{-1} region, corrected using Savitzky-Golay 2nd order differentiation, and vector normalised).

The significance of the differences between tadpoles and spawn from each site/year was determined using one-way ANOVA followed by Tukey's multiple comparison tests.

Supplementary References

1 Movasaghi, Z., Rehman, S. & ur Rehman, D. I. Fourier transform infrared (FTIR) spectroscopy of biological tissues. *Applied Spectroscopy Reviews* **43**, 134-179 (2008).

2 Bellisola, G. & Sorio, C. Infrared spectroscopy and microscopy in cancer research and diagnosis. *Am. J. Cancer Res.* **2**, 1-21 (2012).

3 Cakmak, G., Togan, I. & Severcan, F. 17β-Estradiol induced compositional, structural and functional changes in rainbow trout liver, revealed by FT-IR spectroscopy: a comparative study with nonylphenol. *Aquat. Toxicol.* **77**, 53-63 (2006).

4 Chu, H.-L., Liu, T.-Y. & Lin, S.-Y. Effect of cyanide concentrations on the secondary structures of protein in the crude homogenates of the fish gill tissue. *Aquatic Toxicology* **55**, 171-176, doi:http://dx.doi.org/10.1016/S0166-445X(01)00177-1 (2001).

5 Cakmak, G., Togan, I., Uğuz, C. & Severcan, F. FT-IR spectroscopic analysis of rainbow trout liver exposed to nonylphenol. *Appl Spectrosc* **57**, 835-841 (2003).

6 Podrabsky, J. E., Carpenter, J. F. & Hand, S. C. Survival of water stress in annual fish embryos: dehydration avoidance and egg envelope amyloid fibers. *American Journal of Physiology - Regulatory, Integrative and Comparative Physiology* **280**, R123-R131 (2001).

7 Naumann, D. FT-infrared and FT-Raman spectroscopy in biomedical research. *Applied Spectroscopy Reviews* **36**, 239-298 (2001).

8 Cakmak, G., Togan, I. & Severcan, F. 17*β*-Estradiol induced compositional, structural and functional changes in rainbow trout liver, revealed by FT-IR spectroscopy: a comparative study with nonylphenol. *Aquatic Toxicology* **77**, 53-63 (2006).

9 Chu, H.-L., Liu, T.-Y. & Lin, S.-Y. Effect of cyanide concentrations on the secondary structures of protein in the crude homogenates of the fish gill tissue. *Aquatic Toxicology* **55**, 171-176 (2001).

10 Palaniappan, P. L. R. M. & Vijayasundaram, V. Fourier transform infrared study of protein secondary structural changes in the muscle of *Labeo rohita* due to arsenic intoxication. *Food and Chemical Toxicology* **46**, 3534-3539, doi:10.1016/j.fct.2008.09.001 (2008).

11 Trevisan, J., Angelov, P. P., Carmichael, P. L., Scott, A. D. & Martin, F. L. Extracting biological information with computational analysis of Fourier-transform infrared (FTIR) biospectroscopy datasets: current practices to future perspectives. *Analyst* **137**, 3202-3215, doi:10.1039/c2an16300d (2012).

12 Krafft, C., Steiner, G., Beleites, C. & Salzer, R. Disease recognition by infrared and Raman spectroscopy. *Journal of Biophotonics* **2**, 13-28, doi:10.1002/jbio.200810024 (2009).

13 Sattlecker, M., Baker, R., Stone, N. & Bessant, C. Support vector machine ensembles for breast cancer type prediction from mid-FTIR micro-calcification spectra. *Chemometrics and Intelligent Laboratory Systems* **107**, 363-370, doi:http://dx.doi.org/10.1016/j.chemolab.2011.05.007 (2011).

14 Fernández Pierna, J. A., Volery, P., Besson, R., Baeten, V. & Dardenne, P. Classification of Modified Starches by Fourier Transform Infrared Spectroscopy Using Support Vector Machines. *Journal of Agricultural and Food Chemistry* **53**, 6581-6585, doi:10.1021/jf0501544 (2005).

15 Subasi, A. & Ismail Gursoy, M. EEG signal classification using PCA, ICA, LDA and support vector machines. *Expert Systems with Applications* **37**, 8659-8666, doi:http://dx.doi.org/10.1016/j.eswa.2010.06.065 (2010).

16 Chang, C.-C. & Lin, C.-J. LIBSVM: a library for support vector machines. *ACM Transactions on Intelligent Systems and Technology (TIST)* **2**, 27 (2011).

17 Hsu, C.-W., Chang, C.-C. & Lin, C.-J. (http://www.csie.ntu.edu.tw/~cjlin/papers/guide/guide.pdf, 2003).

18 Huang, C.-L., Liao, H.-C. & Chen, M.-C. Prediction model building and feature selection with support vector machines in breast cancer diagnosis. *Expert Systems with Applications* **34**, 578-587 (2008).
